# Supplementary material for: Vancomycin resistant Enterococci and its associated factors among HIV infected patients on anti-retroviral therapy in Ethiopia
Source: PLoS One. 2021 Jun 24;16(6):e0251727. doi: 10.1371/journal.pone.0251727 (PMC8224944; doi:10.1371/journal.pone.0251727)
Supplement: S1 File — (PDF) [file pone.0251727.s001.pdf]

Annex: Questionnaire (Amharic language version)

መጠየቅ (የአማርኛትርጓሜ)

በአርባምንጭ ዩኒቨርስቲ ህክምናና ጤና ሳይንስ ኮሌጅ

- ይህ ቃለ መጠይቅ የተዘጋጀው “ቫንኮማይስን መድሃኒትን የተላመደ ኢንትሮኮከስ ባክቴሪያ ወረራ (colonization) በአርባ ምንጭ አጠቃላይ ሆስፒታል የጸረ ኤችአይቪ መድሀኒት የሚከታተሉ የኤችአይቪ ቫይረስ በደማቸው ውስጥ ያለባቸው ህሙማን ላይ ያለው ስርጭትና የወረራውን (colonization) ተዛማጅ ምክንያቶች” በሚካሄድ ጥናት ላይ የሚሳተፉ ግለሰቦችን መረጃ ለመሰብሰብ የተዘጋጀ ነው፡፡ ለጥናቱ መሳካት እና ትክክለኛነት ያግዘን ዘንድ ጥያቄዎችን በጥንቃቄ አንብበው ወይም ተረድተው በታማኝነት ያለምንም ፍራቻ እንዲሞሉልን በትህትና እንጠይቃለን፡፡

ኮድ---------------------------

አድራሻ --ስልክ ቁጥር--------------------------

<u>ማሳሰቢያ</u>:- ሁሉም ተገልጋዮች ባለፈዉ አንድ ሳምንት የጸረ-ባክቴሪያ መድሐኒት ያልወሰዱ መሆን ይኖረባቸዋል፡፡

ክፍል 1. አጠቃላይ መረጃ		
ተ.ቁ	ጥያቄዎች	አማራጭ
01	ፆታ	1. ወንድ<br>2. ሴት
02	እድሜ	-------------- ዓመት
03	ነዋሪነቶ	1. ከተማ<br>2. ገጠር
04	የትምህርትደረጃ	1. ያልተማረ/ች<br>2. የመጀመሪያ ደረጃ (1-8)<br>3. ሁለተኛ ደረጃ (9-12)<br>4. ኮሌጅና ከዚያ በላይ

05	የታካሚነት ሁኔታ	1. ተኝቶ ሚታከም<br>2. ተመላላሽ ታካሚ
**ክፍል 2. ኢንትሮኮከስ ባክቴሪያ ወረራውን (Colonization) ሊያስከትሉ የሚችሉ ተዛማጅ (ተጓዳኝ) የጤና ችግሮችን በተመለከተ**		
06	የሄሞግሎቢን መጠን (ከላብራቶሪ መጤት ቅጽ የሚሞላ)	ለወንድ<br>1. ዝቅተኛ (<13.5 g/dl)<br>2. መጠነኛ (13.5-17.5 g/dl)<br>3. ከፍተኛ (>17.5 g/dl)<br>ለሴት<br>4. ዝቅተኛ (<12.0 g/dl)<br>5. መጠነኛ (12.0-15.5 g/dl)<br>6. ከፍተኛ(>15.5 g/dl)
07	ከዚህ በፊት የጸረ-ባክቴሪያ መዲሀኒት ተጠቅመው ሚያውቁ ከሆነ	1. በጭራሽ<br>2. ከ2 ሳምንታት በላይ<br>3. 2 ሳምንታትና ከዛ በታች
08	ባለፉት ስድስት ሳምንታት ሆስፒታል ተኝተው ሚያውቁ ከሆነ	1. አዎ<br>2. የለም
09	ከዚህ በፊት የፕላስቲክ ሽንት ቱቦ ተደርጎሎት ያውቃል?	1. አዎ<br>2. የለም
10	የስኳር ህመም አለብዎት ወይም ኩላሊቶን በጠና ታመው ያውቀሉ?	1. አዎ<br>2. የለም
