# Supplementary material for: Vancomycin resistant Enterococci and its associated factors among HIV infected patients on anti-retroviral therapy in Ethiopia
Source: PLoS One. 2021 Jun 24;16(6):e0251727. doi: 10.1371/journal.pone.0251727 (PMC8224944; doi:10.1371/journal.pone.0251727)
Supplement: S2 File — (DOCX) [file pone.0251727.s002.docx]

**Annex : Questionnaires**

These questionnaires were prepared to determine the incidence of Vancomycin Resistant *Enterococci* and its associated factors among HIV-Infected patients on ART

Code Number ________

Address _________________ _________________ Phone number___________

| **Part 1. Socio-Demographic Data** | | | |
| --- | --- | --- | --- |
| **Sr. no** | **Questions to be asked** | | **Response** |
| **01** | Sex | | - Male 2. Female |
| **02** | Age | | ______________ years old |
| **03** | Place of residence | | - Urban 2. Rural |
| **04** | Educational level | | - Illiterate 3. Secondary (9-12) - Primary (1-8) 4. College and above |
| **05** | Current visiting status | | - In patient 2. Out patient |
| **Part II. Clinical Factors of Study Participants** | | | |
| **06** | | CD4 count (to be filled from laboratory request form) | ----------------(cells/mm^3^) |
| **07** | | Level of hemoglobin (to be filled from laboratory request form) | ---------------- (g/dl) |
| **08** | | Previous antibiotic treatment | - Never - For >2 weeks - For 2 weeks |
| **09** | | History of hospitalization in the last six months | 1.Yes 2.No |
| **10** | | History of previous catheterization? | - Yes 2. No |
| **11** | | Comorbid condition (Diabetes ) | - Yes 2. No |
| **12** | | Comorbid condition (Renal failure) | 1. Yes 2. No |
| **Part III. Laboratory Result Registration Format (Only completed by Principal Investigator)**  Date of sample collection: ____________________ | | | |
| **13** | | Characteristic growth of brown-black colored colonies with dark halo centers on Bile Esculin Azide Agar (BEAA) | - Yes - No |
| **14** | | Catalase test | - Positive 2. Negative |
| **15** | | Gram stains; Gram-positive cocci in pairs or short chains | - Yes 2. No |
| **16** | | Salt tolerance test; growth in brain heart infusion (BHI) broth containing 6.5% NaCl indicated by turbidity. | - Yes - No |
| **17** | | Heat tolerance test (Growth at 45°C); indicated by turbidity. | - Yes - No |
| **18** | | Isolation of *Enterococcus* species : | - Yes - No |
