# Supplementary material for: Vancomycin resistant Enterococci and its associated factors among HIV infected patients on anti-retroviral therapy in Ethiopia
Source: PLoS One. 2021 Jun 24;16(6):e0251727. doi: 10.1371/journal.pone.0251727 (PMC8224944; doi:10.1371/journal.pone.0251727)
Supplement: Attachment — Submitted filename: PONE-D-20-34817_R1_comments.pdf [file pone.0251727.s004.pdf]

PLOS ONE

Vancomycin resistant enterococci and its associated factors among HIV infected patients on anti-Retro viral therapy in Ethiopia

--Manuscript Draft--

**Manuscript Number:**	PONE-D-20-34817R1
**Article Type:**	Research Article
**Full Title:**	Vancomycin resistant enterococci and its associated factors among HIV infected patients on anti-Retro viral therapy in Ethiopia
**Short Title:**	Vancomycin resistant enterococci and associated factors among HIV infected patients
**Corresponding Author:**	Belayneh Regasa Dadi, Ph.D.<br>Arba Minch University<br>Arba Minch, ETHIOPIA
**Keywords:**	Human immunodeficiency virus; Vancomycin Resistant Enterococci
**Abstract:**	Background : The emergence of vancomycin resistant Enterococci (VRE) has alarmed the global community due to its tendency for colonization of the gastrointestinal tract. Human Immunodeficiency Virus (HIV) patients are colonized by vancomycin resistant Enterococci than other groups. The aim of this study was to determine the incidence of vancomycin resistant Enterococci and its associated factors among HIV infected patients on Anti-Retroviral Therapy (ART).<br>Methods : Institution based cross sectional study was conducted among HIV infected patients on ART at from June 1 to August 30, 2020. Socio-demographic and clinical data were collected by pre-tested structured questionnaire. Stool sample was collected and processed by standard microbiological techniques. Kirby Bauer Disc diffusion method was used to perform antimicrobial susceptibility testing. Data were entered by Epi data version 4.6.0.2 and analyzed by SPSS version 25. Bivariable and multivariable logistic regression model was used to analyze the association between dependent and independent variables. P-values in the multivariable analysis, adjusted odds ratio (AOR) and 95% confidence interval (CI) were used to determine the strength of association. P-value $<$ 0.05 was considered as significant.<br>Results : Enterococci spp was isolated on 123/200 (61.50%) patients. Among these isolates, the incidence of vancomycin resistant Enterococci was 11.4% [95% CI: (6.0-17.0)]. Antimicrobial susceptibility patterns against Enterococci showed highest rate of resistance to ampicillin (69.9%). Multidrug resistances were observed in 49.59% of Enterococci isolates. Study participants who had prior antibioticexposurer more than two weeks [AOR=7.35; 95% CI: (1.2144.64)] and hospit alization for the last six months [AOR=5.68; 95% CI: (1.09 29.74)] were significantly associated with vancomycin resistant Enterococci .<br>Conclusions : In our study high incidence of vancomycin resistant Enterococci was found. Previous exposure to antibiotics for more than two weeks and hospitalization for more than six months were significantly associated with vancomycin resistant Enterococci . The isolated Enterococci had variable degrees of resistance to commonly prescribed antibiotics. Therefore, periodic surveillance on antimicrobial resistance pattern, adhering to rational use of antibiotics and implementing infection prevention protocols may reduce colonization by VRE.
**Order of Authors:**	Belayneh Regasa Dadi
	Zerihun Solomon
	Mheret Tesfaye
**Response to Reviewers:**	Point by point response<br>1.Response to editors<br>Comment given: Please ensure that your manuscript meets PLOS ONE's style requirements, including those for file naming. The PLOS ONE style templates can be found at<br>https://journals.plos.org/plosone/s/file?id=wjVg/PLOSOne_formatting_sample_main_bo dy.pdf<br>andhttps://journals.plos.org/plosone/s/file?id=ba62/PLOSOne_formatting_sample_title_

Powered by Editorial Manager® and ProduXion Manager® from Aries Systems Corporation

	authors_affiliations.pdf<br>Response: Comment accepted and corrections were made following the guideline and sample template. (Check for tracking documents where changes made accordingly)<br>Comment given: Please include additional information regarding the survey or questionnaire used in the study and ensure that you have provided sufficient details that others could replicate the analyses. For instance, if you developed a questionnaire as part of this study and it is not under a copyright more restrictive than CC-BY, please include a copy, in both the original language and English, as Supporting Information.<br>Response: Comment accepted and questionnaire (both in local language and English) included as additional information.<br>Comment given: Thank you for stating the following financial disclosure:<br>"The funders had no role in study design, data collection and analysis, decision to publish, or preparation of the manuscript."<br>At this time, please address the following queries:<br>a.Please clarify the sources of funding (financial or material support) for your study. List the grants or organizations that supported your study, including funding received from your institution.<br>b.State what role the funders took in the study. If the funders had no role in your study, please state: “The funders had no role in study design, data collection and analysis, decision to publish, or preparation of the manuscript.”<br>c.If any authors received a salary from any of your funders, please state which authors and which funders.<br>d.If you did not receive any funding for this study, please state: “The authors received no specific funding for this work.”<br>Response: Comment accepted financial disclosure mentioned in the manuscript “The funders had no role in study design, data collection and analysis, decision to publish, or preparation of the manuscript. The authors received no specific funding for this work” (see page 17 paragraph 1)<br>Comment given: We note that you have indicated that data from this study are available upon request. PLOS only allows data to be available upon request if there are legal or ethical restrictions on sharing data publicly. In your revised cover letter, please address the following prompts: a) If there are ethical or legal restrictions on sharing a de-identified data set, please explain them in detail (e.g., data contain potentially identifying or sensitive patient information) and who has imposed them (e.g., an ethics committee). Please also provide contact information for a data access committee, ethics committee, or other institutional body to which data requests may be sent.<br>Response: We have stated that data from this study are available upon request. This is due to Ethical issues (data contain sensitive patient information that cannot be disclosed and can be used for research only). The data can be found upon reasonable request from corresponding author (belayjanimen@gmail.com).<br>Comment given: PLOS requires an ORCID iD for the corresponding author in Editorial Manager on papers submitted after December 6th, 2016. Please ensure that you have an ORCID iD and that it is validated in Editorial Manager. To do this, go to ‘Update my Information’ (in the upper left-hand corner of the main menu), and click on the Fetch/Validate link next to the ORCID field. This will take you to the ORCID site and allow you to create a new iD or authenticate a pre-existing iD in Editorial Manager.<br>Response: Comment accepted.<br>Comment given: Your ethics statement should only appear in the Methods section of your manuscript. If your ethics statement is written in any section besides the Methods, please move it to the Methods section and delete it from any other section. Please ensure that your ethics statement is included in your manuscript, as the ethics statement entered into the online submission form will not be published alongside your manuscript.<br>Response: Comment accepted and ethics section moved to Methods section (see page 8 Ethics approval section)<br>Comment given: We note you have included a table to which you do not refer in the text of your manuscript. Please ensure that you refer to Table 5 in your text; if accepted, production will need this reference to link the reader to the Table.<br>Response: Table 5 is already mentioned on page 11 in line with Table 4 [Table 4 and 5] (on section “Factors associated with vancomycin resistant Enterococcus gut colonization”)<br>Comment given: Please include your tables as part of your main manuscript and remove the individual files. Please note that supplementary tables (should remain/ be

	uploaded) as separate "supporting information" files.<br>Response: Comment accepted and all Tables included in the manuscript (see page 8-12 for Table 1-5)<br>Comment given: Please include captions for your Supporting Information files at the end of your manuscript, and update any in-text citations to match accordingly.<br>Response: Comment accepted.<br>Comment given: The reviewers have commented on the manuscript and raised number of pertinent points that need to be addressed. Discussion section should interpret the results and discuss in the light of current knowledge rather than repeating the results. This applies to Conclusion section abstract too. Please revise the manuscript addressing all reviewer comments.<br>Response: Comment accepted and revisions were made according to comment give by reviewers and see the responses given below:-<br>2.Response to Reviewers<br>Comment given: The language should be improved.<br>Response: We tried to amend and also re-write some parts of the manuscript. Check the manuscript again [please check for Abstract, Introduction, Materials and Methods, Result, Discussion, Conclusion and Limitations of the study sections.]<br>Comment given: The manuscript presents extensive introduction which can be reduced to two or three paragraphs.<br>Response: Comment accepted and Introduction section reduced to three paragraphs on the manuscript (see page 2)<br>Comment given: Statistical tests should be mentioned in the abstract also.<br>Response: Comment accepted and statistical tests added to Abstract section (“Bivariable and multivariable logistic regression model was used to analyze the association between dependent and independent variables. P-values in the multivariable analysis, adjusted odds ratio (AOR) and 95% confidence interval (CI) were used to determine the strength of association. P-value <0.05 was considered as significant.”)<br>Comment given: In methodology, some sentences can be eliminated like the section on antimicrobial susceptibility testing.<br>Response: Comment accepted and antimicrobial susceptibility testing deleted (page 4)<br>Comment given: The name of the institute should be masked.<br>Response: It is impossible to mask the name of the institute where research was conducted. If we mask the name of the institute where the research is conducted, the research will not be trustworthy to the scientific world.<br>Comment given: Discussion needs to be improved. Rather than stating the facts, the results should be compared and justification (or hypotheses) for any differences should be explored.<br>Response: Comment accepted and discussion section revised according to the comments.<br>Comment given: The disk diffusion test alone was used. Explain why MIC was not estimated and species level identification was not performed.<br>Response: Due to limitation of resources and budget, species level identification and MIC were not performed (see page 13 “Limitations of the study” section.)
**Additional Information:**	
**Question**	**Response**
**Financial Disclosure**<br><br>Enter a financial disclosure statement that describes the sources of funding for the work included in this submission. Review the submission guidelines for detailed requirements. View published research articles from *PLOS ONE* for specific examples.<br><br>This statement is required for submission and **will appear in the published article** if	The authors received no specific funding for this work

Powered by Editorial Manager® and ProduXion Manager® from Aries Systems Corporation

the submission is accepted. Please make sure it is accurate.<br><br>**Unfunded studies**<br>Enter: *The author(s) received no specific funding for this work.*<br><br>**Funded studies**<br>Enter a statement with the following details:<br>• Initials of the authors who received each award<br>• Grant numbers awarded to each author<br>• The full name of each funder<br>• URL of each funder website<br>• Did the sponsors or funders play any role in the study design, data collection and analysis, decision to publish, or preparation of the manuscript?<br>• **NO** - Include this sentence at the end of your statement: *The funders had no role in study design, data collection and analysis, decision to publish, or preparation of the manuscript.*<br>• **YES** - Specify the role(s) played.<br><br>* typeset	
**Competing Interests**<br><br>Use the instructions below to enter a competing interest statement for this submission. On behalf of all authors, disclose any competing interests that could be perceived to bias this work—acknowledging all financial support and any other relevant financial or non-financial competing interests.<br><br>This statement **will appear in the published article** if the submission is accepted. Please make sure it is accurate. View published research articles from *PLOS ONE* for specific examples.	he authors have declared that no competing interests exist.

NO authors have competing interests

Enter: *The authors have declared that no competing interests exist.*

Authors with competing interests

Enter competing interest details beginning with this statement:

I have read the journal's policy and the authors of this manuscript have the following competing interests: [insert competing interests here]

* typeset

Ethics Statement

Enter an ethics statement for this submission. This statement is required if the study involved:

- Human participants
- Human specimens or tissue
- Vertebrate animals or cephalopods
- Vertebrate embryos or tissues
- Field research

Write "N/A" if the submission does not require an ethics statement.

General guidance is provided below. Consult the submission guidelines for detailed instructions. **Make sure that all information entered here is included in the Methods section of the manuscript.**

Ethical clearance was obtained from Institutional Review Board (IRB) of Arba Minch University, College of Medicine and Health Sciences, and letter of permission to conduct the study was written to Arba Minch General Hospital. Official permission from Arba Minch General Hospital and an informed written assent/consent from each study participants (assent of those <18 age group from their family or guardian) were obtained.
All information that identifies study subjects were given code numbers and were kept confidential. The purpose of the study was clearly described to the study participants and the specimens collected from the patients were analyzed for the intended purpose only. For each confirmed resistant case, the responsible clinicians of the patient were informed for appropriate management.

Powered by Editorial Manager® and ProduXion Manager® from Aries Systems Corporation

**Format for specific study types**<br><br>**Human Subject Research (involving human participants and/or tissue)**<br>• Give the name of the institutional review board or ethics committee that approved the study<br>• Include the approval number and/or a statement indicating approval of this research<br>• Indicate the form of consent obtained (written/oral) or the reason that consent was not obtained (e.g. the data were analyzed anonymously)<br><br>**Animal Research (involving vertebrate animals, embryos or tissues)**<br>• Provide the name of the Institutional Animal Care and Use Committee (IACUC) or other relevant ethics board that reviewed the study protocol, and indicate whether they approved this research or granted a formal waiver of ethical approval<br>• Include an approval number if one was obtained<br>• If the study involved *non-human primates*, add *additional details* about animal welfare and steps taken to ameliorate suffering<br>• If anesthesia, euthanasia, or any kind of animal sacrifice is part of the study, include briefly which substances and/or methods were applied<br><br>**Field Research**<br><br>Include the following details if this study involves the collection of plant, animal, or other materials from a natural setting:<br>• Field permit number<br>• Name of the institution or relevant body that granted permission	
**Data Availability**<br><br>Authors are required to make all data underlying the findings described fully available, without restriction, and from the time of publication. PLOS allows rare exceptions to address legal and ethical concerns. See the PLOS Data Policy and FAQ for detailed information.	No - some restrictions will apply

A Data Availability Statement describing where the data can be found is required at submission. Your answers to this question constitute the Data Availability Statement and **will be published in the article**, if accepted.<br><br>**Important:** Stating 'data available on request from the author' is not sufficient. If your data are only available upon request, select 'No' for the first question and explain your exceptional situation in the text box.<br><br>Do the authors confirm that all data underlying the findings described in their manuscript are fully available without restriction?	
**Describe where the data may be found in full sentences. If you are copying our sample text, replace any instances of XXX with the appropriate details.**<br><br>• If the data are **held or will be held in a public repository**, include URLs, accession numbers or DOIs. If this information will only be available after acceptance, indicate this by ticking the box below. For example: *All XXX files are available from the XXX database (accession number(s) XXX, XXX.*).<br>• If the data are all contained **within the manuscript and/or Supporting Information files**, enter the following: *All relevant data are within the manuscript and its Supporting Information files.*<br>• If neither of these applies but you are able to provide **details of access elsewhere**, with or without limitations, please do so. For example:<br><br>*Data cannot be shared publicly because of [XXX]. Data are available from the XXX Institutional Data Access / Ethics Committee (contact via XXX) for researchers who meet the criteria for access to confidential data.*<br><br>*The data underlying the results presented in the study are available from (include the name of the third party*	Data cannot be shared publicly because of ethical issues. However the data underlying the results presented in the study are available from corresponding author (belayjanimen@gmail.com) and Institutional Review Board (IRB) committee /Zerihun Zerdo (zedozerihun@gmail.com) on reasonable request.

and contact information or URL).<br>• This text is appropriate if the data are owned by a third party and authors do not have permission to share the data.<br><br>* typeset	
Additional data availability information:	

Manuscript Click here to access/download;Manuscript;Manuscript.docx

Vancomycin resistant *Enterococci* and its associated factors among HIV infected patients on anti-Retro viral therapy in Ethiopia

Belayneh Regasa Dadi[1*], Zerihun Solomon[1], Mheret Tesfaye[1]

1. Department of Medical Microbiology, Arba Minch University, Arba Minch, Ethiopia

*Corresponding author

E-mail: belayjanimen@gmail.com (BRD)

Abstract

Background: The emergence of vancomycin resistant Enterococci (VRE) has alarmed the global community due to its tendency for colonization of the gastrointestinal tract. Human Immunodeficiency Virus (HIV) patients are colonized by vancomycin resistant Enterococci than other groups. The aim of this study was to determine the incidence of vancomycin resistant Enterococci and its associated factors among HIV infected patients on Anti-Retroviral Therapy (ART).

Methods: Institution based cross sectional study was conducted among HIV infected patients on ART at from June 1 to August 30, 2020. Socio-demographic and clinical data were collected by pre-tested structured questionnaire. Stool sample was collected and processed by standard microbiological techniques. Kirby Bauer Disc diffusion method was used to perform antimicrobial susceptibility testing. Data were entered by Epi data version 4.6.0.2 and analyzed by SPSS version 25. Bivariable and multivariable logistic regression model was used to analyze the association between dependent and independent variables. P-values in the multivariable analysis, adjusted odds ratio (AOR) and 95% confidence interval (CI) were used to determine the strength of association. P-value $\leq$0.05 was considered as significant.

Results: *Enterococci* spp was isolated on 123/200 (61.50%) patients. Among these isolates, the incidence of vancomycin resistant Enterococci was 11.4% [95% CI: (6.0-17.0)]. Antimicrobial susceptibility patterns against *Enterococci* showed highest rate of resistance to ampicillin (69.9%). Multidrug resistances were observed in 49.59% of *Enterococci* isolates. Study participants who had prior antibioticexposurer more than two weeks [AOR=7.35; 95% CI: (1.2144.64)] and hospitalization for the last six months [AOR=5.68; 95% CI: (1.09 29.74)] were significantly associated with vancomycin resistant *Enterococci*.

Conclusions: In our study high incidence of vancomycin resistant *Enterococci* was found. Previous exposure to antibiotics for more than two weeks and hospitalization for more than six months were significantly associated with vancomycin resistant *Enterococci*. The isolated *Enterococci* had variable degrees of resistance to commonly prescribed antibiotics. Therefore, periodic surveillance on antimicrobial resistance pattern, adhering to rational use of antibiotics and implementing infection prevention protocols may reduce colonization by VRE.

Keywords: Human Immunodeficiency Virus, Vancomycin Resistant *Enterococci*

Introduction

A major problem with the *Enterococci* is they are very resistant to antibiotics and have ability to survive in harsh environments in community and persist in hospital settings [1]. Because of this, they become important in health facility based settings [2]**.** According to World Health Organization (WHO) report in 2017, vancomycin resistant Enterococci (VRE) is one of the most resistant bacteria in their "Global Priority list of antibiotic-resistant bacteria" [3]. In the same manner, the Center for Disease Control and Prevention (CDC) has classified *Enterococci* among bacteria with a threat level of serious [4]. Currently, VRE are the cause of one-third and one fifth of all health care associated infections in the United States and in some European countries respectively [5].

VRE are known in causing different enterococcal infections such as infective endocarditis, bacteremia, urinary tract infection, intra-abdominal and pelvic infections, surgical wound infections, and very rarely Central nervous system (CNS) infection. Many of these infections originate from intestinal flora of colonized individuals. VRE have different selection pressures for proliferation and rapid expansion of its resistant populations. Furthermore, few options are left for management of diseases caused by VRE, and hence causing increased mortality on infected individuals. VRE now represent approximately one third of *Enterococcus* isolates [6-17].

Asymptomatic VRE gut colonization precedes infection with susceptible hosts. Such susceptible hosts are patients who are exposed to multiple and prolonged courses of antimicrobial agents like Human Immunodeficiency virus (HIV) infected individuals, severely ill, hospitalized for long lengths of stay (LOS), living in a long-term care facility, located in close proximity to another colonized or infected patient, or hospitalized in a room previously occupied by a patient colonized with VRE. Colonization is often obtained by vulnerable hosts in an environment with increased rate of patient colonization with VRE [2,18-26]. The prevalence of VRE was reported in Europe, Asia, Australia, South America and some African countries [1,5,24,26]. However, there is no sufficient data available on the prevalence and risk factors of VRE in developing countries like Ethiopia. Therefore, this study was conducted with the aim of determining the

prevalence of vancomycin resistant enterococci and its associated factors among HIV infected patients on Anti-Retroviral Therapy (ART).

Materials and methods

Study design, period and setting

The study was conducted in Arba Minch General Hospital (Arba Minch, Ethiopia) from June 1 to August 30, 2020. The hospital has ART clinic where it provides different services for HIV-infected patients. The total population of Arba Minch town from 2007 central statistical agency (CSA) census report was 74,879, of whom 39,208 were men and 35,671 women [27]. The inclusion criteria was all HIV infected patients on ART during the study period. Exclusion criteria were HIV infected patients who don't have Parent or guardian assent if they are <18 years old and those HIV infected patients who were critically ill and unable to respond.

Sampling technique

Systematic random sampling technique was used to select the study participants. Based on the 2020 three months (June-August, 2020) data obtained during COVID-19 pandemic from Arba Minch General Hospital ART clinic, N=366 (N= total number of study participants) HIV infected patients has visited the ART clinic. Assuming the same number of HIV- infected patients for the study period (June-August, 2020) and taking sample size of 200, the kth value (k=N/n=366/2002) is found to be 2. The first one is selected with lottery method from 1st and 2nd patients and found to be 2nd patient. Then every 2nd patient was included.

Dependent variable was VRE gut colonization and independent variables were: Age, Sex, CD4 count, Level of hemoglobin, Previous antibiotic treatment for >2 weeks, Current visiting status (Inpatient or Outpatient), History of hospitalization in the last six months, Urinary catheterization, Co-morbid conditions (diabetes and renal failure).

Data collection and laboratory processing

A pretested well designed structured questionnaire was used to collect data from the study participants. The questionnaire was designed by reviewing different literatures and prepared in English and Amharic languages.

Upon the arrival of each study participant at ART clinic, written assent/consent was obtained. Socio-demographic and clinical data were collected by two nurses using a pretested well designed structured questionnaire through face-to-face interview. Moreover, recent CD4 count and hemoglobin level of respondents have been taken from ART clinic logbook.

Isolation and Identification of *Enterococci*

Patients were instructed to collect about 2gm of the faecal specimen in a sterile wide-mouth screw capped container labeled with the unique sample number, date, and time of collection. The collected stool specimens were transported to Arba Minch University, College of Medicine and Health Sciences, Microbiology and Parasitology laboratory.

Immediately after delivery, the transported stool specimens was streaked on Bile Esculin Azide agar (BEAA) (Park Scientific Limited, 24 Low Farm Place, Moulton Park, Northampton, NN3 6HY) and incubated for 24 hours at 37°C. Plates were observed for appearance of characteristic growth with brown-black colored colonies in the medium and dark halo centers. Typical characteristic colonies were selected randomly for characterization and presumptive identification of *Enterococci* by Gram stains, Catalase test, Salt tolerance test and Heat tolerance test [29].

The antimicrobial susceptibility testing was performed using Kirby Bauer disc diffusion method according to Clinical Laboratory Standards Institute guidelines (CLSI) [30]. After a pure colony was obtained, a loop full of bacteria were taken, transferred to a tube containing 5 ml of sterile normal saline (0.85% NaCl) and mixed gently until it formed a homogenous suspension. The turbidity of the suspension was determined by comparison with 0.5 McFarland standards. A sterile swab was dipped into the suspension, and excess suspension was removed by pressing the swab against the wall of the tube. The entire surface of Muller Hinton Agar plate was uniformly flooded with suspensions and allowed to dry for about 3-5 minutes. The antimicrobial impregnated disks were placed by using sterile forceps at least 24 mm away from each other to avoid the overlapping zone of inhibition. The disks were placed on agar plates and allowed to stand for 30 minutes to dissolve the antibiotics in the media [16]. The plates were then inverted and incubated at 37°c for 16-18 hours and inhibition zone was measured using a ruler.

In the case of vancomycin, the plates were inverted and incubated at 37°c, and held a full 16-18 hours for accurate detection of resistance. Zones were examined using transmitted light; the presence of a haze or any growth within the zone of inhibition indicates resistance for vancomycin. Grades of susceptibility pattern were recognized as sensitive, intermediate and resistant by comparison of zone of inhibition according to the 2018 CLSI guidelines [30].

Antimicrobial susceptibility patterns of *Enterococci* were also assessed against the following antibiotic discs: Penicillin (10 IU), Ampicillin (10 µg), Tetracycline (30µg), Doxycycline (30µg), Ciprofloxacin (5µg), Vancomycin (30 µg), Erythromycin (15µg) and Chloramphenicol (30µg). Interpretations of results were made according to CLSI (30). Antibiotics were selected based on CLSI recommendation, local availability (in health facility) and feasibility (cost and method of antimicrobial susceptibility test).

Data Quality Assurance

A pretest was done on 5 % (n=10) HIV infected individuals. Quality control measures were implementing throughout the entire process of data collection and laboratory work. Training was given for data and sample collectors concerning on the research objective, data collection tools, sample collection procedures, and infection prevention protocols to be taken related with COVID-19 Pandemic. Standard Operating Procedures (SOP) were prepared and implemented strictly. All culture media were prepared following the manufacturer's instruction and sterility of the culture media was tested by incubating 5% of the batch at 35–37°C overnight for evaluation of possible contamination. Standard control strains *E. faecalis* ATCC 29212 and *Staphylococcus aureus* ATCC 25923 were used as a positive control.

Statistical analysis

Data were checked for its completeness, entered by Epi data version 4.6.0.2 software, and analyzed by SPSS version 25. The fitness of the model was checked by Hosmer-Lemeshow goodness of fit test. Bivariable and multivariable logistic regression model was used to analyze the association between dependent and independent variables. Those variables with P-value < 0.25 in bivariate analysis were considered as candidate for further multivariable analysis. P-values in the multivariable analysis, adjusted odds ratio (AOR) and 95% confidence interval (CI)

were used to determine the strength of association. P-value <0.05 was considered as statistically significant.

Ethics approval and consent to participate

Ethical clearance was obtained from Institutional Review Board (IRB) of Arba Minch University, College of Medicine and Health Sciences, and letter of permission to conduct the study was written to Arba Minch General Hospital. Official permission from Arba Minch General Hospital and an informed written assent/consent from each study participants (assent of those <18 age group from their family or guardian) were obtained.

All information that identifies study subjects were given code numbers and were kept confidential. The purpose of the study was clearly described to the study participants and the specimens collected from the patients were analyzed for the intended purpose only. For each confirmed resistant case, the responsible clinicians of the patient were informed for appropriate management.

Results

Socio-demographic characteristics of study participants

A total of 200 study participants were enrolled in this study. Among these the ratio of male to female was 1:1. The mean age and standard deviation was 38.68 ± 10.97 ranging from 18-67 years. More than three fourth of the study participants 154 (77.0%) came from urban settings (**Table 1**).

Table 1. Socio-demographic characteristics of study participants attending ART Clinic at Arba Minch General Hospital, Southern Ethiopia October 2020 (N=200)

**Variables**	**Frequency**	**Percentage (%)**
**Sex**		
Males	100	50.0
Females	100	50.0
**Age**		
≤20	12	6.0

21-30	30	15.0
31-40	78	39.0
41-50	47	23.5
≥51	33	16.5
**Residence**		
Rural	46	23.0
Urban	154	77.0
**Educational Status**		
Unable to read and write	17	8.5
Able to read and write	42	21.0
Primary (1-8)	62	31.0
Secondary (9-12)	62	31.0
College and above	17	8.5

Clinical characteristics of study participants

The clinical data showed that majority of the participants 187 (93.5%) were outpatients and 168 (84.0%) of the participants had CD4 count >350. Less than half of the respondents 83 (41.5%) had low hemoglobin level and 94 (47.0%) had history of previous antibiotic use for <2 weeks. Majority of the study participants 174 (87.0%) didn't have previous history of hospitalization in the last six months and 180 (90.0%) didn't have history of previous catheterization. Likewise, majority of the respondents 191 (95.5%) and 193 (96.5%) didn't have Diabetes Mellitus and Renal failure respectively (**Table 2**).

Table 2. Clinical Characteristics of Study Participants Attending ART Clinic at Arba Minch General Hospital, Southern Ethiopia October 2020 (N=200)

**Visiting Status**	**Frequency**	**Percentage**
Outpatient	187	93.5
Inpatient	13	6.5
**CD_4+ Count**		
≤350	32	16.0
>350	168	84.0
**Hemoglobin Level**		

Low	83	41.5
Normal	111	55.5
High	6	3.0
**Previous Antibiotic Treatment**		
Never	76	38.0
≥2 weeks	30	15.0
<2 weeks	94	47.0
**History of hospitalization in the last six months**		
No	174	87.0
Yes	26	13.0
**History of previous catheterization**		
No	180	90.0
Yes	20	10.0
**Comorbid condition (Diabetes)**		
No	191	95.5
Yes	9	4.5
**Comorbid condition (Renal failure)**		
No	193	96.5
Yes	7	3.5

Incidence of vancomycin resistant *Enterococcus*

From the total of 200 study participants, *Enterococci* species was isolated from 123 (61.50%) patients. Among these isolates, 14 (11.4%) were vancomycin resistant (95% CI: 6.0-17.0%) (**Figure 1**).

Figure 1. Incidence of vancomycin resistant *Enterococci* among clients attending ART Clinic at Arba Minch General Hospital, Southern Ethiopia October 2020 (N=123)

Antimicrobial resistance profile of isolated *Enterococci*

Among 123 *Enterococci* isolates tested for commonly prescribed antimicrobial agents the highest rate of resistance was observed against ampicillin in which more than two thirds 86 (69.9%) of the isolates were resistant (**Figure 2**).

Figure 2. Antimicrobial resistance patterns of isolated *Enterococcus* spp among clients attending ART Clinic at Arba Minch General Hospital, Southern Ethiopia October 2020 (n=123)

Multidrug resistances (MDR) were observed in slightly less than half 61 (49.59%) of *Enterococci* isolates and less than one tenth 11 (8.94%) of the isolates were resistant to all antimicrobials tested. All VRE isolates were MDR (**Table 3**).

Table 3. Profile of Multidrug Resistance Pattern of VRE isolates among clients Attending ART Clinic at Arba Minch General Hospital, Southern Ethiopia October 2020 (n=14)

Resistance rate	Combination of Antibiotics	No. (%) of isolates tested
R4	G, P, TTC, MAC	1 (7.14)
R5	G, P, TTC, F, MAC	2 (14.28)
R6	G, P, TTC, F, MAC, PH	11 (78.58)

Key; G-glycopeptides (vancomycin), P-penicillins (ampicillin and/or penicillin), TTC-tetracyclines (tetracycline and/or doxycycline), MAC-macrolides (erythromycin), F-fluoroquinolones (ciprofloxacin), PH-phenicols (chloramphenicol), and R4-R6 Number of categories of antibiotics resistance from 4 to 6, respectively

Factors associated with vancomycin resistant *Enterococcus* gut colonization

Factors independently associated with VRE gut colonization on bivariable analysis, and fit into a multivariable logistic regression model were sex, visiting status, history of catheterization, previous antibiotics treatment, history of hospitalization in the last six months and presence of Diabetes Mellitus (**Table 4 and 5**).

Table 4. Bivariable Logistic Regression analysis on factors affecting vancomycin resistant *Enterococci* among clients attending ART Clinic at Arba Minch General Hospital, Southern Ethiopia October 2020

**Characteristics**	**Categories**	**VRE gut colonization**		**COR**	**P-value**
		**Yes, N (%)**	**No, N (%)**		
Sex	Females	4 (6.8)	55 (93.2)	**0.39**	**0.133***
	Males	10 (15.6)	54 (84.4)	1	
Residence	Urban	11 (11.6)	84 (88.4)	1.09	0.899
	Rural	3 (10.7)	25 (89.3)	1	
Visiting status	Inpatient	3 (30.0)	7 (70.0)	**3.97**	**0.069***
	Outpatient	11 (9.7)	102 (90.3)	1	
CD4 count	≤350	2 (11.8)	15 (88.2)	1.04	0.957
	>350	12 (11.3)	94 (88.7)	1	
Previous Antibiotic Treatment	Never	2 (4.9)	39 (95.1)	1	
	≥2 weeks	8 (40.0)	12 (60.0)	**13.00**	**0.003***
	<2 weeks	4 (6.5)	58 (93.5)	1.34	0.739
History of hospitalization in the last six months	Yes	6 (37.5)	10 (62.5)	**7.43**	**0.002***
	No	8 (7.5)	99 (92.5)	1	
History of previous catheterization	Yes	4 (25.0)	12 (75.0)	**3.23**	**0.078***
	No	10 (9.3)	97 (90.7)	1	
Diabetes Mellitus	Yes	2 (28.6)	5 (71.4)	**3.48**	**0.163***
	No	12 (10.3)	104 (89.7)	1	
*Variables which passed bivariable logistic regression analysis at cut off value <0.25					

Table 5. Multivariable Logistic Regression analysis on factors affecting vancomycin resistant *Enterococci* among clients attending ART Clinic at Arba Minch General Hospital, Southern Ethiopia October 2020

**Characteristics**	**Categories**	**VRE isolates**		**AOR (95%CI)**	**P-value**
		**Yes, N (%)**	**No, N (%)**		
Sex	Females	4 (6.8)	55 (93.2)	0.27 (0.06-1.25)	0.093
	Males	10 (15.6)	54 (84.4)	1	
Visiting status	Inpatient	3 (30.0)	7 (70.0)	1.13 (0.15-8.43)	0.904
	Outpatient	11 (9.7)	102 (90.3)	1	
Previous Antibiotic Treatment	Never	2 (4.9)	39 (95.1)	1	
	≥2 weeks	8 (40.0)	12 (60.0)	**7.35 (1.21-44.64)**	**0.030****
	<2 weeks	4 (6.5)	58 (93.5)	1.08 (0.17-6.80)	0.936
History of hospitalization in the last six months	Yes	6 (37.5)	10 (62.5)	**5.68 (1.09-29.74)**	**0.040****
	No	8 (7.5)	99 (92.5)	1	
History of previous catheterization	Yes	4 (25.0)	12 (75.0)	1.38 (0.27-7.03)	0.701
	No	10 (9.3)	97 (90.7)	1	
Diabetes Mellitus	Yes	2 (28.6)	5 (71.4)	1.81 (0.21 - 15.36)	0.588
	No	12 (10.3)	104 (89.7)	1	
**Significant in multivariable logistic regression at p-value <0.05					

Discussion

The hasty emergence and the increasing incidence of colonization with VRE have become challenging healthcare problems that have caused serious concern to health care providers and health authorities [2]. In the present study, the prevalence of VRE among HIV infected patients was 11.4% (95% CI: 6.0-17.0%). This prevalence rate was consistent with other studies conducted in South Africa (14.5%) [21], West Amhara, Ethiopia (7.7%) [2], Addis Ababa, Ethiopia (6.7%) [31] and Gondar, Northwest Ethiopia (7.8%) [18]. However, the prevalence of

VRE in our study is lower than the report from Ireland (44.1%) [4], Germany (26.1%) [32] Brazil (23.4%) [1], India (19.6%) [33], Saud Arabia (17.3%) [5] and Iraq (46.4%) [17]. The lower prevalence in the present study might be due to the variation in study settings in which most of the previous countries have been using oral and intravenous vancomycin massively for human diseases [34,35], and variation in study participants where the previous studies participants were hospitalized patients and critically ill patients in ICU who were frequently exposed to different antibiotics and experienced insertion of external devices like catheter [7]. On the other hand, the prevalence in our study is higher than studies conducted in USA (4.7%) [36], Nigeria (4.07%) [37] and Ethiopia (5.9%) [16]. The gradual increase and clonal expansion in the prevalence of VRE might have contributed to this higher prevalence [32,38].

Enterococci isolates showed various resistances to the tested antibiotics; namely, 69.9% to ampicillin, 54.5% to penicillin, 49.6% to erythromycin, 59.3% to tetracycline, 28.5% to ciprofloxacin and 21.1% to chloramphenicol. These findings are comparable with studies conducted in India 64.9% [33] and Ethiopia 66.7% [39] for ampicillin; Ethiopia 64.9% [40], 68% [39] for tetracycline; and Brazil 45.7% [1] and Ethiopia 42.7% [9] 53.3% [31] for erythromycin. However, the resistance profiles in our study is lower than previous studies in India for tested antibiotics 75.9% for penicillin, 84.5% for tetracycline, 95.5% for ciprofloxacin, 92.1% for erythromycin and 42.3% for chloramphenicol [33]; Uganda 69.4% [15] and Ethiopia 77.3% [41] for tetracycline; and Iraq (85.7%) [17], Uganda (72%) [15] and Ethiopia 63.2% [41] erythromycin; These lower drug resistance patterns might be due to variations in sample size, methodology and study participants where most of the participants in the previous studies were hospitalized patients who were taking different antibiotics that might have been contributed for emergence of high rate of drug resistance.

On the other hand, the antibiotic resistance profile in our study is higher than studies conducted in Iran 41.2% [42], Brazil 0% [1], Uganda 1.4% [15] and Ethiopia 36% [41] for ampicillin; Brazil 32.6% [1] and Ethiopia 37.7% [2] for tetracycline; and Brazil 10.9% [1] for chloramphenicol. The high drug resistance patterns might be due to overuse or misuse of antibiotics and inappropriate antibiotics prescription which is very common practice in Ethiopia. Additional reasons for high drug resistance might be extensive antibiotics use of antibiotics for agricultural purpose, mutation and gene transfer among bacteria [4].

Moreover, the present study also showed that 49.6% of *Enterococci* isolates were multidrug resistant. This result is lower than the findings in Iraq (85.7%) [17] and Ethiopia 80.8% [41]. In

contrary, the result is higher than the finding in Ethiopia 29.5% [16]. The discrepancy of the result might be due to variation in geographical distribution of strain, trend and frequency of antibiotic prescription, community drug usage practice.

Our study showed that HIV patients who were previously exposed to antibiotics for more than two weeks were seven times more likely to be colonized with VRE as compared with HIV patients who never exposed to antibiotics previously [AOR=7.35; 95% CI: (1.21-44.64); P-value= 0.030]. This result is in agreement with other studies conducted in Brazil [1], South Korea [43], Egypt [44], and Ethiopia [2]. The reason might be a prior exposure to antibiotics for a prolonged duration can cause VRE colonization due to the fact that the antibiotics exert selective pressure to *Enterococci* and alter the competing microbial flora in the GI tract allowing VRE to predominate as evidenced by other studies [7,34].

The present study showed that HIV patients who had history of hospitalization for the last six months were six times more likely to be colonized with VRE as compared with HIV patients who didn't have history of hospitalization for the last six months [AOR=5.68; 95% CI: (1.09-29.74); P-value = 0.040]. The finding is consistent with previous studies done in USA [36], South Korea [43] and Ethiopia [2]. The reason might be VRE have been isolated from virtually every object within patient rooms since they are intrinsically resistant to several commonly used antibiotics in hospital and have ability to acquire resistance genes. Besides, they are ubiquitous in their presence and have high survivability on dry surfaces, thereby causing high VRE transmission rates within healthcare facilities [7,9].

Limitations of the study

The isolated *Enterococci* were not identified to species level; molecular characterization and MIC were done due to resource limitation and budget constraints.

Conclusions

In our study high incidence of vancomycin resistant Enterococci was found. Previous exposure to antibiotics for more than two weeks and previous hospitalization for more than six months were significant factors for vancomycin resistant enterococci gut colonization. The study also

showed that the isolated *Enterococci* had variable degrees of resistance to commonly prescribed antibiotics.

Therefore periodic surveillance on antimicrobial resistance pattern of *Enterococcus* species is important for proper treatment, health professionals should strictly follow infection prevention protocols and further studies should be conducted on species identification and molecular characterization of *Enterococci*.

List of abbreviations

ART: Anti-Retroviral Therapy **AOR**: Adjusted odd ratio **BEAA**: Bile Esculin Azide agar **CDC**: Center for Disease Control **CI**: Confidence Interval **CLSI**: Clinical Laboratory Standards Institute guidelines **CSA**: Central statistical agency **HAI**: Hospital Acquired Infections **HIV**: Human Immunodeficiency Virus **MDR**: Multi Drug Resistant **SOP**: Standard Operating Procedures **VRE**: Vancomycin Resistance *Enterococcus* **WHO**: World Health Organization

Acknowledgments

The authors would like to thank those who were involved in this research.

Authors' contributions

ZS, BRD, and MT carried out proposal development, data collection, data analysis and drafted the manuscript. All authors read and approved the final manuscript.

Competing interests

The authors declare that they have no competing interests

Funding: The funders had no role in study design, data collection and analysis, decision to publish, or preparation of the manuscript. The authors received no specific funding for this work.

References

1. Batistão DW da F, Gontijo-Filho PP, Conceição N, Oliveira AG de, Ribas RM (2012). Risk factors for vancomycin-resistant enterococci colonisation in critically ill patients. *Mem Inst Oswaldo Cruz.* 107(1):57–63.
2. Agegne M, Abera B, Derbie A, Yismaw G, Shiferaw MB (2018). Magnitude of Vancomycin-Resistant Enterococci (VRE) Colonization among HIV-Infected Patients Attending ART Clinic in West Amhara Government Hospitals. *Int J Microbiol.* 1–7.
3. Tacconelli E (2017). Global Priority List of Antibiotic-Resistant Bacteria to Guide Research, Discovery, and Development of New Antibiotics. WHO.
4. Ventola CL (2015). The Antibiotic Resistance Crisis: Part 1: Causes and Threats. *Pharm Ther*. 40(4):277.
5. Alotaibi FE, Bukhari EE (2017). Emergence of Vancomycin-resistant Enterococci at a Teaching Hospital, Saudi Arabia. *Chin Med J (Engl).* 130(3):340–6.
6. Emaneini M, Hosseinkhani F, Jabalameli F, Nasiri MJ, Dadashi M, Pouriran R, et al (2016). Prevalence of vancomycin-resistant Enterococcus in Iran: a systematic review and meta-analysis. *Eur J Clin Microbiol Infect Dis*. 35(9):1387–92.
7. Alevizakos M, Gaitanidis A, Nasioudis D, Tori K, Flokas ME, Mylonakis E (2017). Colonization With Vancomycin-Resistant Enterococci and Risk for Bloodstream Infection Among Patients With Malignancy: A Systematic Review and Meta-Analysis. *Open Forum Infect Dis* [Internet]. [cited 2019 Nov 5];4(1).
8. Susan L Fraser CJD, Robert A Salata (2018). Vancomycin Resistant Enterococcal Infections: Background, Pathophysiology, Epidemiology. *Medscape.* 97(4):223–7.
9. Murray PR, Rosenthal KS, Pfaller MA (2016). Medical microbiology. 8th edition. Philadelphia, PA: *Elsevier*.
10. Nilsson O (2012). Vancomycin resistant enterococci in farm animals – occurrence and importance. *Infect Ecol Epidemiol*. 2(1):16959.
11. Sood S, Malhotra M, Das BK, Kapil A (2008). Enterococcal infections & antimicrobial resistance. *Indian J Med Res*. 128(2):111–21.
12. Crank C, O'Driscoll T (2015). Vancomycin-resistant enterococcal infections: epidemiology, clinical manifestations, and optimal management. *Infect Drug Resist.* 217.
13. Carroll KC, Hobden JA, Miller S, Morse SA, Mietzner TA, Detrick B, *et al* (2016). Jawetz, Melnick & Adelberg's medical microbiology. 27 th edition. *New York: Mcgraw-Hill Education*.

14. Faron ML, Ledeboer NA, Buchan BW (2016). Resistance Mechanisms, Epidemiology, and Approaches to Screening for Vancomycin-Resistant Enterococcus in the Health Care Setting. Kraft CS, editor. *J Clin Microbiol.* 54(10):2436–47.
15. Kateete DP, Edolu M, Kigozi E, Kisukye J, Baluku H, Mwiine FN, *et al* (2019). Species, antibiotic susceptibility profiles and van gene frequencies among enterococci isolated from patients at Mulago National Referral Hospital in Kampala, Uganda. *BMC Infect Dis*. 19(1):486.
16. Ali S, Alemayehu M, Dagnew M, Gebrecherkos T (2018). Vancomycin-Resistant Enterococci and Its Associated Risk Factors among HIV-Positive and -Negative Clients Attending Dessie Referral Hospital, Northeast Ethiopia. *Int J Microbiol.* 1–9.
17. Al-Dahmoshi HOM, Rabeea HWS, Aridhee ASA, Al-Khafaji NSK, Al-Allak MH, Lazm AM, *et al* (2019). Phenotypic Investigation of Vancomycin, Teicoplanin and Linezolid Resistance Among Enterococcus spp. Isolated from Children Diarrhea. *J Pure Appl Microbiol.* 13(1):531–6.
18. Abebe W, Endris M, Tiruneh M, Moges F. Prevalence of vancomycin resistant Enterococci and associated risk factors among clients with and without HIV in Northwest Ethiopia: a cross-sectional study. *BMC Public Health.* 2014 Dec;14(1):185.
19. Amberpet R, Sistla S, Parija SC, Rameshkumar R (2018). Risk factors for intestinal colonization with vancomycin resistant enterococci' A prospective study in a level III pediatric intensive care unit. *J Lab Physicians*. 10(1):89–94.
20. Axelrad JE, Lebwohl B, Cuaresma E, Cadwell K, Green PHR, Freedberg DE (2018). Gut colonization with vancomycin-resistant Enterococcus and risk for subsequent enteric infection. *Gut Pathog.* 10(1):28.
21. Tatsing Foka FE, Kumar A, Ateba CN (2018). Emergence of vancomycin-resistant enterococci in South Africa: Implications for public health. *South Afr J Sci* [Internet]. [cited 2019 Dec 3];114(9/10).
22. Orsi GB, Ciorba V (2013). Vancomycin resistant enterococci healthcare associated infections. *Ann Ig.* 25(10):485–92.
23. Rameshkumar MR, Arunagirinathan N (2018). Drug-Resistant Bacterial Infections in HIV Patients. *Intech Open*. 98(5).
24. Kwobah C, Ali I, Koskei P, Chumba D, Emonyi W, Mwangi A, *et al* (2015) Antibiotic Sensitivity Patterns Among Postmortem Bacterial Isolates From HIV-Infected Patients in Western Kenya: A Cross-sectional Descriptive Study. *J Dis Glob Health*. 3:73–82.

25. Chinnambedu RS, Marimuthu RR, Sunil SS, Amrose P, Ramachandran V, Pachamuthu B (2020). Changing antibiotic resistance profile of Staphylococcus aureus isolated from HIV patients (2012–2017) in Southern India. *J Infect Public Health.*13(1):75–9.
26. Remschmidt C, Schröder C, Behnke M, Gastmeier P, Geffers C, Kramer TS (2018). Continuous increase of vancomycin resistance in enterococci causing nosocomial infections in Germany − 10 years of surveillance. *Antimicrob Resist Infect Control*. 7(1):54.
27. Census 2007 Tables: Southern Peoples, Nations and Nationalities Region Archived November 13, 2012, at the Way back Machine. Tables 2.1, and 3.4.
28. Naing L, Winn T, Rusli BN (2006). Medical Statistics Practical Issues in Calculating the Sample Size for Prevalence Studies. *Archives of Orofacial Sciences* 1: 9-14.
29. Bile Esculin Azide Agar ISO Formato 30 Plates [Internet]. [cited 2020 Jan 24].
30. CLSI (2018) Clinical and Laboratory Standards Institute, Performance Standards for Antimicrobial Susceptibility Testing, CLSI, Wayne, PA, USA, 28th edition.
31. Ferede ZT, Tullu KD, Derese SG, Yeshanew AG (2018). Prevalence and antimicrobial susceptibility pattern of Enterococcus species isolated from different clinical samples at Black Lion Specialized Teaching Hospital, Addis Ababa, Ethiopia. *BMC Res Note*s 11(793). DOI: 10.1186/s13104-018-3898-0
32. Markwart R, Willrich N, Haller S, Noll I, Koppe U, Werner G, *et al* (2019). The rise in vancomycin-resistant Enterococcus faecium in Germany: data from the German Antimicrobial Resistance Surveillance (ARS). *Antimicrob Resist Infect Control*. 8(1):1–11.
33. Deshpande VR, Karmarkar MG, Mehta PR (2013). Prevalence of multidrug-resistant enterococci in a tertiary care hospital in Mumbai, India. *J Infect Dev Ctries*. 7(02):155–8.
34. Flokas ME, Karageorgos SA, Detsis M, Alevizakos M, Mylonakis E (2017). Vancomycin-resistant enterococci colonisation, risk factors and risk for infection among hospitalised paediatric patients: a systematic review and meta-analysis. *Int J Antimicrob Agents*. 49(5):565–72.
35. Cattoir V, Leclercq R (2013). Twenty-five years of shared life with vancomycin-resistant enterococci: is it time to divorce? *J Antimicrob Chemother*. 68(4):731–42.
36. Achenbach C, Flores E, Ferrell P, Pitrak D, Weber SG (2006). Prevalence of and Risk Factors for Colonization With Vancomycin-Resistant Enterococcus Among Human Immunodeficiency Virus–Positive Outpatients. *Infect Control Hosp Epidemiol*. 27(1):102–4.

37. Ekuma AE, Oduyebo OO, Efunshile AM, Konig B (2016). Surveillance for vancomycin resistant enterococci in a tertiary institution in south western Nigeria. *Afr J Infect Dis.* 10(2):121–6.
38. Swissnoso, Buetti N, Wassilew N, Rion V, Senn L, Gardiol C, *et al* (2019) Emergence of vancomycin-resistant enterococci in Switzerland: a nation-wide survey. *Antimicrob Resist Infect Control.* 8(1):16.
39. Yilema A, Moges F, Tadele S, Endris M, Kassu A, Abebe W, *et al* (2017) Isolation of enterococci, their antimicrobial susceptibility patterns and associated factors among patients attending at the University of Gondar Teaching Hospital. *BMC Infect Dis.* 17(1):276.
40. Abamecha A, Wondafrash B, Abdissa A (2015). Antimicrobial resistance profile of Enterococcus species isolated from intestinal tracts of hospitalized patients in Jimma, Ethiopia. *BMC Res Notes*. 8(1):213.
41. Toru M, Beyene G, Kassa T, Gizachew Z, Howe R, Yeshitila B (2018). Prevalence and phenotypic characterization of Enterococcus species isolated from clinical samples of pediatric patients in Jimma University Specialized Hospital, south west Ethiopia. *BMC Res Notes*. 11(1):281.
42. Anvarinejad M, Pouladfar G, Japoni A, Bolandparvaz S, Satiary Z, Mardaneh J (2016). Diabetic Foot Infections: Antibiotic Susceptibility Patterns and Determination of Antibiotic Cross-Resistance in Clinical Isolates of Enterococcus Species During 2012 - 2014 in Shiraz, Iran. *Arch Pediatr Infect Dis*. 5(2).
43. Kee SY, Park CW, Lee JE, Kwon YJ, Pyo HJ, *et al.*(2012) Healthcare-associated risk factors of vancomycin-resistant Enterococci colonization among outpatients undergoing hemodialysis. *Jpn J Infect Dis*. 65(1):57–60.
44. Moemen D, Tawfeek D, Badawy W (2015). Healthcare-associated vancomycin resistant Enterococcus faecium infections in the Mansoura University Hospitals intensive care units, Egypt. *Braz J Microbiol.* 46(3):777–83.

Manuscript Click here to access/download;Manuscript;Manuscript.docx

Vancomycin resistant *Enterococci* and its associated factors among HIV infected patients on anti-Retro viral therapy in Ethiopia

Belayneh Regasa Dadi[1*], Zerihun Solomon[1], Mheret Tesfaye[1]

1. Department of Medical Microbiology, Arba Minch University, Arba Minch, Ethiopia

*Corresponding author

E-mail: belayjanimen@gmail.com (BRD)

Abstract

Background: The emergence of vancomycin resistant Enterococci (VRE) has alarmed the global community due to its tendency for colonization of the gastrointestinal tract. Human Immunodeficiency Virus (HIV) patients are colonized by vancomycin resistant Enterococci than other groups. The aim of this study was to determine the incidence of vancomycin resistant Enterococci and its associated factors among HIV infected patients on Anti-Retroviral Therapy (ART).

Methods: Institution based cross sectional study was conducted among HIV infected patients on ART at from June 1 to August 30, 2020. Socio-demographic and clinical data were collected by pre-tested structured questionnaire. Stool sample was collected and processed by standard microbiological techniques. Kirby Bauer Disc diffusion method was used to perform antimicrobial susceptibility testing. Data were entered by Epi data version 4.6.0.2 and analyzed by SPSS version 25. Bivariable and multivariable logistic regression model was used to analyze the association between dependent and independent variables. P-values in the multivariable analysis, adjusted odds ratio (AOR) and 95% confidence interval (CI) were used to determine the strength of association. P-value $\leq$0.05 was considered as significant.

Results: *Enterococci* spp was isolated on 123/200 (61.50%) patients. Among these isolates, the incidence of vancomycin resistant Enterococci was 11.4% [95% CI: (6.0-17.0)]. Antimicrobial susceptibility patterns against *Enterococci* showed highest rate of resistance to ampicillin (69.9%). Multidrug resistances were observed in 49.59% of *Enterococci* isolates. Study participants who had prior antibioticexposurer more than two weeks [AOR=7.35; 95% CI: (1.2144.64)] and hospitalization for the last six months [AOR=5.68; 95% CI: (1.09 29.74)] were significantly associated with vancomycin resistant *Enterococci*.

Conclusions: In our study high incidence of vancomycin resistant *Enterococci* was found. Previous exposure to antibiotics for more than two weeks and hospitalization for more than six months were significantly associated with vancomycin resistant *Enterococci*. The isolated *Enterococci* had variable degrees of resistance to commonly prescribed antibiotics. Therefore, periodic surveillance on antimicrobial resistance pattern, adhering to rational use of antibiotics and implementing infection prevention protocols may reduce colonization by VRE.

Keywords: Human Immunodeficiency Virus, Vancomycin Resistant *Enterococci*

Introduction

A major problem with the *Enterococci* is they are very resistant to antibiotics and have ability to survive in harsh environments in community and persist in hospital settings [1]. Because of this, they become important in health facility based settings [2]**.** According to World Health Organization (WHO) report in 2017, vancomycin resistant Enterococci (VRE) is one of the most resistant bacteria in their "Global Priority list of antibiotic-resistant bacteria" [3]. In the same manner, the Center for Disease Control and Prevention (CDC) has classified *Enterococci* among bacteria with a threat level of serious [4]. Currently, VRE are the cause of one-third and one fifth of all health care associated infections in the United States and in some European countries respectively [5].

VRE are known in causing different enterococcal infections such as infective endocarditis, bacteremia, urinary tract infection, intra-abdominal and pelvic infections, surgical wound infections, and very rarely Central nervous system (CNS) infection. Many of these infections originate from intestinal flora of colonized individuals. VRE have different selection pressures for proliferation and rapid expansion of its resistant populations. Furthermore, few options are left for management of diseases caused by VRE, and hence causing increased mortality on infected individuals. VRE now represent approximately one third of *Enterococcus* isolates [6-17].

Asymptomatic VRE gut colonization precedes infection with susceptible hosts. Such susceptible hosts are patients who are exposed to multiple and prolonged courses of antimicrobial agents like Human Immunodeficiency virus (HIV) infected individuals, severely ill, hospitalized for long lengths of stay (LOS), living in a long-term care facility, located in close proximity to another colonized or infected patient, or hospitalized in a room previously occupied by a patient colonized with VRE. Colonization is often obtained by vulnerable hosts in an environment with increased rate of patient colonization with VRE [2,18-26]. The prevalence of VRE was reported in Europe, Asia, Australia, South America and some African countries [1,5,24,26]. However, there is no sufficient data available on the prevalence and risk factors of VRE in developing countries like Ethiopia. Therefore, this study was conducted with the aim of determining the

prevalence of vancomycin resistant enterococci and its associated factors among HIV infected patients on Anti-Retroviral Therapy (ART).

Materials and methods

Study design, period and setting

The study was conducted in Arba Minch General Hospital (Arba Minch, Ethiopia) from June 1 to August 30, 2020. The hospital has ART clinic where it provides different services for HIV-infected patients. The total population of Arba Minch town from 2007 central statistical agency (CSA) census report was 74,879, of whom 39,208 were men and 35,671 women [27]. The inclusion criteria was all HIV infected patients on ART during the study period. Exclusion criteria were HIV infected patients who don't have Parent or guardian assent if they are <18 years old and those HIV infected patients who were critically ill and unable to respond.

Sampling technique

Systematic random sampling technique was used to select the study participants. Based on the 2020 three months (June-August, 2020) data obtained during COVID-19 pandemic from Arba Minch General Hospital ART clinic, N=366 (N= total number of study participants) HIV infected patients has visited the ART clinic. Assuming the same number of HIV- infected patients for the study period (June-August, 2020) and taking sample size of 200, the kth value (k=N/n=366/2002) is found to be 2. The first one is selected with lottery method from 1st and 2nd patients and found to be 2nd patient. Then every 2nd patient was included.

Dependent variable was VRE gut colonization and independent variables were: Age, Sex, CD4 count, Level of hemoglobin, Previous antibiotic treatment for >2 weeks, Current visiting status (Inpatient or Outpatient), History of hospitalization in the last six months, Urinary catheterization, Co-morbid conditions (diabetes and renal failure).

Data collection and laboratory processing

A pretested well designed structured questionnaire was used to collect data from the study participants. The questionnaire was designed by reviewing different literatures and prepared in English and Amharic languages.

Upon the arrival of each study participant at ART clinic, written assent/consent was obtained. Socio-demographic and clinical data were collected by two nurses using a pretested well designed structured questionnaire through face-to-face interview. Moreover, recent CD4 count and hemoglobin level of respondents have been taken from ART clinic logbook.

Isolation and Identification of *Enterococci*

Patients were instructed to collect about 2gm of the faecal specimen in a sterile wide-mouth screw capped container labeled with the unique sample number, date, and time of collection. The collected stool specimens were transported to Arba Minch University, College of Medicine and Health Sciences, Microbiology and Parasitology laboratory.

Immediately after delivery, the transported stool specimens was streaked on Bile Esculin Azide agar (BEAA) (Park Scientific Limited, 24 Low Farm Place, Moulton Park, Northampton, NN3 6HY) and incubated for 24 hours at 37°C. Plates were observed for appearance of characteristic growth with brown-black colored colonies in the medium and dark halo centers. Typical characteristic colonies were selected randomly for characterization and presumptive identification of *Enterococci* by Gram stains, Catalase test, Salt tolerance test and Heat tolerance test [29].

The antimicrobial susceptibility testing was performed using Kirby Bauer disc diffusion method according to Clinical Laboratory Standards Institute guidelines (CLSI) [30]. After a pure colony was obtained, a loop full of bacteria were taken, transferred to a tube containing 5 ml of sterile normal saline (0.85% NaCl) and mixed gently until it formed a homogenous suspension. The turbidity of the suspension was determined by comparison with 0.5 McFarland standards. A sterile swab was dipped into the suspension, and excess suspension was removed by pressing the swab against the wall of the tube. The entire surface of Muller Hinton Agar plate was uniformly flooded with suspensions and allowed to dry for about 3-5 minutes. The antimicrobial impregnated disks were placed by using sterile forceps at least 24 mm away from each other to avoid the overlapping zone of inhibition. The disks were placed on agar plates and allowed to stand for 30 minutes to dissolve the antibiotics in the media [16]. The plates were then inverted and incubated at 37°c for 16-18 hours and inhibition zone was measured using a ruler.

In the case of vancomycin, the plates were inverted and incubated at 37°c, and held a full 16-18 hours for accurate detection of resistance. Zones were examined using transmitted light; the presence of a haze or any growth within the zone of inhibition indicates resistance for vancomycin. Grades of susceptibility pattern were recognized as sensitive, intermediate and resistant by comparison of zone of inhibition according to the 2018 CLSI guidelines [30].

Antimicrobial susceptibility patterns of *Enterococci* were also assessed against the following antibiotic discs: Penicillin (10 IU), Ampicillin (10 µg), Tetracycline (30µg), Doxycycline (30µg), Ciprofloxacin (5µg), Vancomycin (30 µg), Erythromycin (15µg) and Chloramphenicol (30µg). Interpretations of results were made according to CLSI (30). Antibiotics were selected based on CLSI recommendation, local availability (in health facility) and feasibility (cost and method of antimicrobial susceptibility test).

Data Quality Assurance

A pretest was done on 5 % (n=10) HIV infected individuals. Quality control measures were implementing throughout the entire process of data collection and laboratory work. Training was given for data and sample collectors concerning on the research objective, data collection tools, sample collection procedures, and infection prevention protocols to be taken related with COVID-19 Pandemic. Standard Operating Procedures (SOP) were prepared and implemented strictly. All culture media were prepared following the manufacturer's instruction and sterility of the culture media was tested by incubating 5% of the batch at 35–37°C overnight for evaluation of possible contamination. Standard control strains *E. faecalis* ATCC 29212 and *Staphylococcus aureus* ATCC 25923 were used as a positive control.

Statistical analysis

Data were checked for its completeness, entered by Epi data version 4.6.0.2 software, and analyzed by SPSS version 25. The fitness of the model was checked by Hosmer-Lemeshow goodness of fit test. Bivariable and multivariable logistic regression model was used to analyze the association between dependent and independent variables. Those variables with P-value < 0.25 in bivariate analysis were considered as candidate for further multivariable analysis. P-values in the multivariable analysis, adjusted odds ratio (AOR) and 95% confidence interval (CI)

were used to determine the strength of association. P-value <0.05 was considered as statistically significant.

Ethics approval and consent to participate

Ethical clearance was obtained from Institutional Review Board (IRB) of Arba Minch University, College of Medicine and Health Sciences, and letter of permission to conduct the study was written to Arba Minch General Hospital. Official permission from Arba Minch General Hospital and an informed written assent/consent from each study participants (assent of those <18 age group from their family or guardian) were obtained.

All information that identifies study subjects were given code numbers and were kept confidential. The purpose of the study was clearly described to the study participants and the specimens collected from the patients were analyzed for the intended purpose only. For each confirmed resistant case, the responsible clinicians of the patient were informed for appropriate management.

Results

Socio-demographic characteristics of study participants

A total of 200 study participants were enrolled in this study. Among these the ratio of male to female was 1:1. The mean age and standard deviation was 38.68 ± 10.97 ranging from 18-67 years. More than three fourth of the study participants 154 (77.0%) came from urban settings (**Table 1**).

Table 1. Socio-demographic characteristics of study participants attending ART Clinic at Arba Minch General Hospital, Southern Ethiopia October 2020 (N=200)

**Variables**	**Frequency**	**Percentage (%)**
**Sex**		
Males	100	50.0
Females	100	50.0
**Age**		
≤20	12	6.0

21-30	30	15.0
31-40	78	39.0
41-50	47	23.5
≥51	33	16.5
**Residence**		
Rural	46	23.0
Urban	154	77.0
**Educational Status**		
Unable to read and write	17	8.5
Able to read and write	42	21.0
Primary (1-8)	62	31.0
Secondary (9-12)	62	31.0
College and above	17	8.5

Clinical characteristics of study participants

The clinical data showed that majority of the participants 187 (93.5%) were outpatients and 168 (84.0%) of the participants had CD4 count >350. Less than half of the respondents 83 (41.5%) had low hemoglobin level and 94 (47.0%) had history of previous antibiotic use for <2 weeks. Majority of the study participants 174 (87.0%) didn't have previous history of hospitalization in the last six months and 180 (90.0%) didn't have history of previous catheterization. Likewise, majority of the respondents 191 (95.5%) and 193 (96.5%) didn't have Diabetes Mellitus and Renal failure respectively (**Table 2**).

Table 2. Clinical Characteristics of Study Participants Attending ART Clinic at Arba Minch General Hospital, Southern Ethiopia October 2020 (N=200)

**Visiting Status**	**Frequency**	**Percentage**
Outpatient	187	93.5
Inpatient	13	6.5
**CD_4+ Count**		
≤350	32	16.0
>350	168	84.0
**Hemoglobin Level**		

Low	83	41.5
Normal	111	55.5
High	6	3.0
**Previous Antibiotic Treatment**		
Never	76	38.0
≥2 weeks	30	15.0
<2 weeks	94	47.0
**History of hospitalization in the last six months**		
No	174	87.0
Yes	26	13.0
**History of previous catheterization**		
No	180	90.0
Yes	20	10.0
**Comorbid condition (Diabetes)**		
No	191	95.5
Yes	9	4.5
**Comorbid condition (Renal failure)**		
No	193	96.5
Yes	7	3.5

Incidence of vancomycin resistant *Enterococcus*

From the total of 200 study participants, *Enterococci* species was isolated from 123 (61.50%) patients. Among these isolates, 14 (11.4%) were vancomycin resistant (95% CI: 6.0-17.0%) (**Figure 1**).

Figure 1. Incidence of vancomycin resistant *Enterococci* among clients attending ART Clinic at Arba Minch General Hospital, Southern Ethiopia October 2020 (N=123)

Antimicrobial resistance profile of isolated *Enterococci*

Among 123 *Enterococci* isolates tested for commonly prescribed antimicrobial agents the highest rate of resistance was observed against ampicillin in which more than two thirds 86 (69.9%) of the isolates were resistant (**Figure 2**).

Figure 2. Antimicrobial resistance patterns of isolated *Enterococcus* spp among clients attending ART Clinic at Arba Minch General Hospital, Southern Ethiopia October 2020 (n=123)

Multidrug resistances (MDR) were observed in slightly less than half 61 (49.59%) of *Enterococci* isolates and less than one tenth 11 (8.94%) of the isolates were resistant to all antimicrobials tested. All VRE isolates were MDR (**Table 3**).

Table 3. Prof[illegible]of Multidrug Resistance Pattern of VRE isolates among clients Attending ART Clinic at Arba Minch General Hospital, Southern Ethiopia October 2020 (n=14)

Resistance rate	Combination of Antibiotics	No. (%) of isolates tested
R4	G, P, TTC, MAC	1 (7.14)
R5	G, P, TTC, F, MAC	2 (14.28)
R6	G, P, TTC, F, MAC, PH	11 (78.58)

Key; G-glycopeptides (vancomycin), P-penicillins (ampicillin and/or penicillin), TTC-tetracyclines (tetracycline and/or doxycycline), MAC-macrolides (erythromycin), F-fluoroquinolones (ciprofloxacin), PH-phenicols (chloramphenicol), and R4-R6 Number of categories of antibiotics resistance from 4 to 6, respectively

Factors associated with vancomycin resistant *Enterococcus* gut colonization

Factors independently associated with VRE gut colonization on bivariable analysis, and fit into a multivariable logistic regression model were sex, visiting status, history of catheterization, previous antibiotics treatment, history of hospitalization in the last six months and presence of Diabetes Mellitus (**Table 4 and 5**).

Table 4. Bivariable Logistic Regression analysis on factors affecting vancomycin resistant *Enterococci* among clients attending ART Clinic at Arba Minch General Hospital, Southern Ethiopia October 2020

**Characteristics**	**Categories**	**VRE gut colonization**		**COR**	**P-value**
		**Yes, N (%)**	**No, N (%)**		
Sex	Females	4 (6.8)	55 (93.2)	**0.39**	**0.133***
	Males	10 (15.6)	54 (84.4)	1	
Residence	Urban	11 (11.6)	84 (88.4)	1.09	0.899
	Rural	3 (10.7)	25 (89.3)	1	
Visiting status	Inpatient	3 (30.0)	7 (70.0)	**3.97**	**0.069***
	Outpatient	11 (9.7)	102 (90.3)	1	
CD4 count	≤350	2 (11.8)	15 (88.2)	1.04	0.957
	>350	12 (11.3)	94 (88.7)	1	
Previous Antibiotic Treatment	Never	2 (4.9)	39 (95.1)	1	
	≥2 weeks	8 (40.0)	12 (60.0)	**13.00**	**0.003***
	<2 weeks	4 (6.5)	58 (93.5)	1.34	0.739
History of hospitalization in the last six months	Yes	6 (37.5)	10 (62.5)	**7.43**	**0.002***
	No	8 (7.5)	99 (92.5)	1	
History of previous catheterization	Yes	4 (25.0)	12 (75.0)	**3.23**	**0.078***
	No	10 (9.3)	97 (90.7)	1	
Diabetes Mellitus	Yes	2 (28.6)	5 (71.4)	**3.48**	**0.163***
	No	12 (10.3)	104 (89.7)	1	
*Variables which passed bivariable logistic regression analysis at cut off value <0.25					

Table 5. Multivariable Logistic Regression analysis on factors affecting vancomycin resistant *Enterococci* among clients attending ART Clinic at Arba Minch General Hospital, Southern Ethiopia October 2020

Characteristics	Categories	VRE isolates		AOR (95%CI)	P-value
		**Yes, N (%)**	**No, N (%)**		
Sex	Females	4 (6.8)	55 (93.2)	0.27 (0.06-1.25)	0.093
	Males	10 (15.6)	54 (84.4)	1	
Visiting status	Inpatient	3 (30.0)	7 (70.0)	1.13 (0.15-8.43)	0.904
	Outpatient	11 (9.7)	102 (90.3)	1	
Previous Antibiotic Treatment	Never	2 (4.9)	39 (95.1)	1	
	≥2 weeks	8 (40.0)	12 (60.0)	**7.35 (1.21-44.64)**	**0.030****
	<2 weeks	4 (6.5)	58 (93.5)	1.08 (0.17-6.80)	0.936
History of hospitalization in the last six months	Yes	6 (37.5)	10 (62.5)	**5.68 (1.09-29.74)**	**0.040****
	No	8 (7.5)	99 (92.5)	1	
History of previous catheterization	Yes	4 (25.0)	12 (75.0)	1.38 (0.27-7.03)	0.701
	No	10 (9.3)	97 (90.7)	1	
Diabetes Mellitus	Yes	2 (28.6)	5 (71.4)	1.81 (0.21 - 15.36)	0.588
	No	12 (10.3)	104 (89.7)	1	
**Significant in multivariable logistic regression at p-value <0.05					

Discussion

The hasty emergence and the increasing incidence of colonization with VRE have become challenging healthcare problems that have caused serious concern to health care providers and health authorities [2]. In the present study, the prevalence of VRE among HIV infected patients was 11.4% (95% CI: 6.0-17.0%). This prevalence rate was consistent with other studies conducted in South Africa (14.5%) [21], West Amhara, Ethiopia (7.7%) [2], Addis Ababa, Ethiopia (6.7%) [31] and Gondar, Northwest Ethiopia (7.8%) [18]. However, the prevalence of

VRE in our study is lower than the report from Ireland (44.1%) [4], Germany (26.1%) [32] Brazil (23.4%) [1], India (19.6%) [33], Saud Arabia (17.3%) [5] and Iraq (46.4%) [17]. The lower prevalence in the present study might be due to the variation in study settings in which most of the previous countries have been using oral and intravenous vancomycin massively for human diseases [34,35], and variation in study participants where the previous studies participants were hospitalized patients and critically ill patients in ICU who were frequently exposed to different antibiotics and experienced insertion of external devices like catheter [7]. On the other hand, the prevalence in our study is higher than studies conducted in USA (4.7%) [36], Nigeria (4.07%) [37] and Ethiopia (5.9%) [16]. The gradual increase and clonal expansion in the prevalence of VRE might have contributed to this higher prevalence [32,38].

Enterococci isolates showed various resistances to the tested antibiotics; namely, 69.9% to ampicillin, 54.5% to penicillin, 49.6% to erythromycin, 59.3% to tetracycline, 28.5% to ciprofloxacin and 21.1% to chloramphenicol. These findings are comparable with studies conducted in India 64.9% [33] and Ethiopia 66.7% [39] for ampicillin; Ethiopia 64.9% [40], 68% [39] for tetracycline; and Brazil 45.7% [1] and Ethiopia 42.7% [9] 53.3% [31] for erythromycin. However, the resistance profiles in our study is lower than previous studies in India for tested antibiotics 75.9% for penicillin, 84.5% for tetracycline, 95.5% for ciprofloxacin, 92.1% for erythromycin and 42.3% for chloramphenicol [33]; Uganda 69.4% [15] and Ethiopia 77.3% [41] for tetracycline; and Iraq (85.7%) [17], Uganda (72%) [15] and Ethiopia 63.2% [41] erythromycin; These lower drug resistance patterns might be due to variations in sample size, methodology and study participants where most of the participants in the previous studies were hospitalized patients who were taking different antibiotics that might have been contributed for emergence of high rate of drug resistance.

On the other hand, the antibiotic resistance profile in our study is higher than studies conducted in Iran 41.2% [42], Brazil 0% [1], Uganda 1.4% [15] and Ethiopia 36% [41] for ampicillin; Brazil 32.6% [1] and Ethiopia 37.7% [2] for tetracycline; and Brazil 10.9% [1] for chloramphenicol. The high drug resistance patterns might be due to overuse or misuse of antibiotics and inappropriate antibiotics prescription which is very common practice in Ethiopia. Additional reasons for high drug resistance might be extensive antibiotics use of antibiotics for agricultural purpose, mutation and gene transfer among bacteria [4].

Moreover, the present study also showed that 49.6% of *Enterococci* isolates were multidrug resistant. This result is lower than the findings in Iraq (85.7%) [17] and Ethiopia 80.8% [41]. In

contrary, the result is higher than the finding in Ethiopia 29.5% [16]. The discrepancy of the result might be due to variation in geographical distribution of strain, trend and frequency of antibiotic prescription, community drug usage practice.

Our study showed that HIV patients who were previously exposed to antibiotics for more than two weeks were seven times more likely to be colonized with VRE as compared with HIV patients who never exposed to antibiotics previously [AOR=7.35; 95% CI: (1.21-44.64); P-value= 0.030]. This result is in agreement with other studies conducted in Brazil [1], South Korea [43], Egypt [44], and Ethiopia [2]. The reason might be a prior exposure to antibiotics for a prolonged duration can cause VRE colonization due to the fact that the antibiotics exert selective pressure to *Enteroc[illegible]i* and alter the competing microbial flora in the GI tract allowing VRE to predominate as evidenced by other studies [7,34].

The present study showed that HIV patients who had history of hospitalization for the last six months were six times more likely to be colonized with VRE as compared with HIV patients who didn't have history of hospitalization for the last six months [AOR=5.68; 95% CI: (1.09-29.74); P-value = 0.040]. The finding is consistent with previous studies done in USA [36], South Korea [43] and Ethiopia [2]. The reason might be VRE have been isolated from virtually every object within patient rooms since they are intrinsically resistant to several commonly used antibiotics in hospital and have ability to acquire resistance genes. Besides, they are ubiquitous in their presence and have high survivability on dry surfaces, thereby causing high VRE transmission rates within healthcare facilities [7,9].

Limitations of the study

The isolated *Enterococci* were not identified to species level; molecular characterization and MIC wer[illegible]ne due to resource limitation and budget constraints.

Conclusions

In our study high incidence of vancomycin resistant Enterococci was found. Previous exposure to antibiotics for more than two weeks and previous hospitalization for more than six months were significant factors for vancomycin resistant enterococci gut colonization. The study also

showed that the isolated *Enterococci* had variable degrees of resistance to commonly prescribed antibiotics.

Therefore periodic surveillance on antimicrobial resistance pattern of *Enterococcus* species is important for proper treatment, health professionals should strictly follow infection prevention protocols and further studies should be conducted on species identification and molecular characterization of *Enterococci*.

List of abbreviations

ART: Anti-Retroviral Therapy **AOR**: Adjusted odd ratio **BEAA**: Bile Esculin Azide agar **CDC**: Center for Disease Control **CI**: Confidence Interval **CLSI**: Clinical Laboratory Standards Institute guidelines **CSA**: Central statistical agency **HAI**: Hospital Acquired Infections **HIV**: Human Immunodeficiency Virus **MDR**: Multi Drug Resistant **SOP**: Standard Operating Procedures **VRE**: Vancomycin Resistance *Enterococcus* **WHO**: World Health Organization

Acknowledgments

The authors would like to thank those who were involved in this research.

Authors' contributions

ZS, BRD, and MT carried out proposal development, data collection, data analysis and drafted the manuscript. All authors read and approved the final manuscript.

Competing interests

The authors declare that they have no competing interests

Funding: The funders had no role in study design, data collection and analysis, decision to publish, or preparation of the manuscript. The authors received no specific funding for this work.

References

1. Batistão DW da F, Gontijo-Filho PP, Conceição N, Oliveira AG de, Ribas RM (2012). Risk factors for vancomycin-resistant enterococci colonisation in critically ill patients. *Mem Inst Oswaldo Cruz.* 107(1):57–63.
2. Agegne M, Abera B, Derbie A, Yismaw G, Shiferaw MB (2018). Magnitude of Vancomycin-Resistant Enterococci (VRE) Colonization among HIV-Infected Patients Attending ART Clinic in West Amhara Government Hospitals. *Int J Microbiol.* 1–7.
3. Tacconelli E (2017). Global Priority List of Antibiotic-Resistant Bacteria to Guide Research, Discovery, and Development of New Antibiotics. WHO.
4. Ventola CL (2015). The Antibiotic Resistance Crisis: Part 1: Causes and Threats. *Pharm Ther*. 40(4):277.
5. Alotaibi FE, Bukhari EE (2017). Emergence of Vancomycin-resistant Enterococci at a Teaching Hospital, Saudi Arabia. *Chin Med J (Engl).* 130(3):340–6.
6. Emaneini M, Hosseinkhani F, Jabalameli F, Nasiri MJ, Dadashi M, Pouriran R, et al (2016). Prevalence of vancomycin-resistant Enterococcus in Iran: a systematic review and meta-analysis. *Eur J Clin Microbiol Infect Dis*. 35(9):1387–92.
7. Alevizakos M, Gaitanidis A, Nasioudis D, Tori K, Flokas ME, Mylonakis E (2017). Colonization With Vancomycin-Resistant Enterococci and Risk for Bloodstream Infection Among Patients With Malignancy: A Systematic Review and Meta-Analysis. *Open Forum Infect Dis* [Internet]. [cited 2019 Nov 5];4(1).
8. Susan L Fraser CJD, Robert A Salata (2018). Vancomycin Resistant Enterococcal Infections: Background, Pathophysiology, Epidemiology. *Medscape*. 97(4):223–7.
9. Murray PR, Rosenthal KS, Pfaller MA (2016). Medical microbiology. 8th edition. Philadelphia, PA: *Elsevier*.
10. Nilsson O (2012). Vancomycin resistant enterococci in farm animals – occurrence and importance. *Infect Ecol Epidemiol*. 2(1):16959.
11. Sood S, Malhotra M, Das BK, Kapil A (2008). Enterococcal infections & antimicrobial resistance. *Indian J Med Res*. 128(2):111–21.
12. Crank C, O'Driscoll T (2015). Vancomycin-resistant enterococcal infections: epidemiology, clinical manifestations, and optimal management. *Infect Drug Resist.* 217.
13. Carroll KC, Hobden JA, Miller S, Morse SA, Mietzner TA, Detrick B, *et al* (2016). Jawetz, Melnick & Adelberg's medical microbiology. 27 th edition. *New York: Mcgraw-Hill Education*.

14. Faron ML, Ledeboer NA, Buchan BW (2016). Resistance Mechanisms, Epidemiology, and Approaches to Screening for Vancomycin-Resistant Enterococcus in the Health Care Setting. Kraft CS, editor. *J Clin Microbiol.* 54(10):2436–47.
15. Kateete DP, Edolu M, Kigozi E, Kisukye J, Baluku H, Mwiine FN, *et al* (2019). Species, antibiotic susceptibility profiles and van gene frequencies among enterococci isolated from patients at Mulago National Referral Hospital in Kampala, Uganda. *BMC Infect Dis*. 19(1):486.
16. Ali S, Alemayehu M, Dagnew M, Gebrecherkos T (2018). Vancomycin-Resistant Enterococci and Its Associated Risk Factors among HIV-Positive and -Negative Clients Attending Dessie Referral Hospital, Northeast Ethiopia. *Int J Microbiol.* 1–9.
17. Al-Dahmoshi HOM, Rabeea HWS, Aridhee ASA, Al-Khafaji NSK, Al-Allak MH, Lazm AM, *et al* (2019). Phenotypic Investigation of Vancomycin, Teicoplanin and Linezolid Resistance Among Enterococcus spp. Isolated from Children Diarrhea. *J Pure Appl Microbiol.* 13(1):531–6.
18. Abebe W, Endris M, Tiruneh M, Moges F. Prevalence of vancomycin resistant Enterococci and associated risk factors among clients with and without HIV in Northwest Ethiopia: a cross-sectional study. *BMC Public Health.* 2014 Dec;14(1):185.
19. Amberpet R, Sistla S, Parija SC, Rameshkumar R (2018). Risk factors for intestinal colonization with vancomycin resistant enterococci' A prospective study in a level III pediatric intensive care unit. *J Lab Physicians*. 10(1):89–94.
20. Axelrad JE, Lebwohl B, Cuaresma E, Cadwell K, Green PHR, Freedberg DE (2018). Gut colonization with vancomycin-resistant Enterococcus and risk for subsequent enteric infection. *Gut Pathog.* 10(1):28.
21. Tatsing Foka FE, Kumar A, Ateba CN (2018). Emergence of vancomycin-resistant enterococci in South Africa: Implications for public health. *South Afr J Sci* [Internet]. [cited 2019 Dec 3];114(9/10).
22. Orsi GB, Ciorba V (2013). Vancomycin resistant enterococci healthcare associated infections. *Ann Ig.* 25(10):485–92.
23. Rameshkumar MR, Arunagirinathan N (2018). Drug-Resistant Bacterial Infections in HIV Patients. *Intech Open*. 98(5).
24. Kwobah C, Ali I, Koskei P, Chumba D, Emonyi W, Mwangi A, *et al* (2015) Antibiotic Sensitivity Patterns Among Postmortem Bacterial Isolates From HIV-Infected Patients in Western Kenya: A Cross-sectional Descriptive Study. *J Dis Glob Health*. 3:73–82.

25. Chinnambedu RS, Marimuthu RR, Sunil SS, Amrose P, Ramachandran V, Pachamuthu B (2020). Changing antibiotic resistance profile of Staphylococcus aureus isolated from HIV patients (2012–2017) in Southern India. *J Infect Public Health.*13(1):75–9.
26. Remschmidt C, Schröder C, Behnke M, Gastmeier P, Geffers C, Kramer TS (2018). Continuous increase of vancomycin resistance in enterococci causing nosocomial infections in Germany − 10 years of surveillance. *Antimicrob Resist Infect Control*. 7(1):54.
27. Census 2007 Tables: Southern Peoples, Nations and Nationalities Region Archived November 13, 2012, at the Way back Machine. Tables 2.1, and 3.4.
28. Naing L, Winn T, Rusli BN (2006). Medical Statistics Practical Issues in Calculating the Sample Size for Prevalence Studies. *Archives of Orofacial Sciences* 1: 9-14.
29. Bile Esculin Azide Agar ISO Formato 30 Plates [Internet]. [cited 2020 Jan 24].
30. CLSI (2018) Clinical and Laboratory Standards Institute, Performance Standards for Antimicrobial Susceptibility Testing, CLSI, Wayne, PA, USA, 28th edition.
31. Ferede ZT, Tullu KD, Derese SG, Yeshanew AG (2018). Prevalence and antimicrobial susceptibility pattern of Enterococcus species isolated from different clinical samples at Black Lion Specialized Teaching Hospital, Addis Ababa, Ethiopia. *BMC Res Note*s 11(793). DOI: 10.1186/s13104-018-3898-0
32. Markwart R, Willrich N, Haller S, Noll I, Koppe U, Werner G, *et al* (2019). The rise in vancomycin-resistant Enterococcus faecium in Germany: data from the German Antimicrobial Resistance Surveillance (ARS). *Antimicrob Resist Infect Control*. 8(1):1–11.
33. Deshpande VR, Karmarkar MG, Mehta PR (2013). Prevalence of multidrug-resistant enterococci in a tertiary care hospital in Mumbai, India. *J Infect Dev Ctries*. 7(02):155–8.
34. Flokas ME, Karageorgos SA, Detsis M, Alevizakos M, Mylonakis E (2017). Vancomycin-resistant enterococci colonisation, risk factors and risk for infection among hospitalised paediatric patients: a systematic review and meta-analysis. *Int J Antimicrob Agents*. 49(5):565–72.
35. Cattoir V, Leclercq R (2013). Twenty-five years of shared life with vancomycin-resistant enterococci: is it time to divorce? *J Antimicrob Chemother*. 68(4):731–42.
36. Achenbach C, Flores E, Ferrell P, Pitrak D, Weber SG (2006). Prevalence of and Risk Factors for Colonization With Vancomycin-Resistant Enterococcus Among Human Immunodeficiency Virus–Positive Outpatients. *Infect Control Hosp Epidemiol*. 27(1):102–4.

37. Ekuma AE, Oduyebo OO, Efunshile AM, Konig B (2016). Surveillance for vancomycin resistant enterococci in a tertiary institution in south western Nigeria. *Afr J Infect Dis.* 10(2):121–6.
38. Swissnoso, Buetti N, Wassilew N, Rion V, Senn L, Gardiol C, *et al* (2019) Emergence of vancomycin-resistant enterococci in Switzerland: a nation-wide survey. *Antimicrob Resist Infect Control.* 8(1):16.
39. Yilema A, Moges F, Tadele S, Endris M, Kassu A, Abebe W, *et al* (2017) Isolation of enterococci, their antimicrobial susceptibility patterns and associated factors among patients attending at the University of Gondar Teaching Hospital. *BMC Infect Dis.* 17(1):276.
40. Abamecha A, Wondafrash B, Abdissa A (2015). Antimicrobial resistance profile of Enterococcus species isolated from intestinal tracts of hospitalized patients in Jimma, Ethiopia. *BMC Res Notes*. 8(1):213.
41. Toru M, Beyene G, Kassa T, Gizachew Z, Howe R, Yeshitila B (2018). Prevalence and phenotypic characterization of Enterococcus species isolated from clinical samples of pediatric patients in Jimma University Specialized Hospital, south west Ethiopia. *BMC Res Notes*. 11(1):281.
42. Anvarinejad M, Pouladfar G, Japoni A, Bolandparvaz S, Satiary Z, Mardaneh J (2016). Diabetic Foot Infections: Antibiotic Susceptibility Patterns and Determination of Antibiotic Cross-Resistance in Clinical Isolates of Enterococcus Species During 2012 - 2014 in Shiraz, Iran. *Arch Pediatr Infect Dis*. 5(2).
43. Kee SY, Park CW, Lee JE, Kwon YJ, Pyo HJ, *et al.*(2012) Healthcare-associated risk factors of vancomycin-resistant Enterococci colonization among outpatients undergoing hemodialysis. *Jpn J Infect Dis*. 65(1):57–60.
44. Moemen D, Tawfeek D, Badawy W (2015). Healthcare-associated vancomycin resistant Enterococcus faecium infections in the Mansoura University Hospitals intensive care units, Egypt. *Braz J Microbiol.* 46(3):777–83.

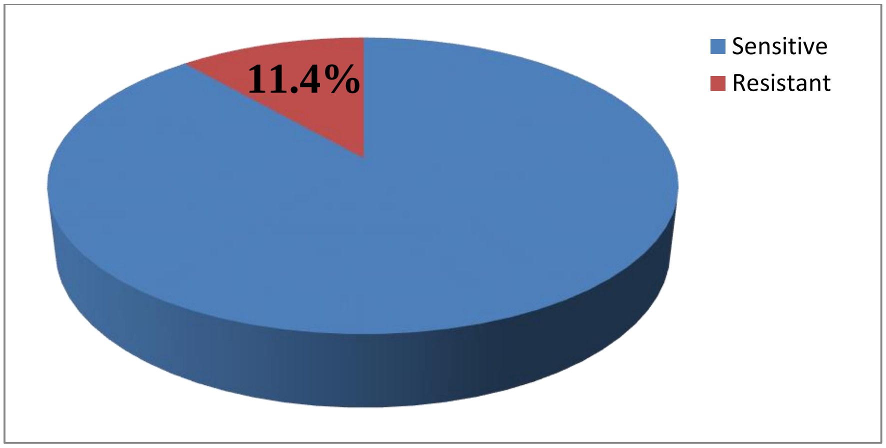

Figure 1. Incidence of Vancomycin Resistant *Enterococci* among Clients Attending ART Clinic at Arba Minch General Hospital, Southern Ethiopia October 2020 (N=123)

Figure 2 Click here to access/download;Figure;Figure 2.docx

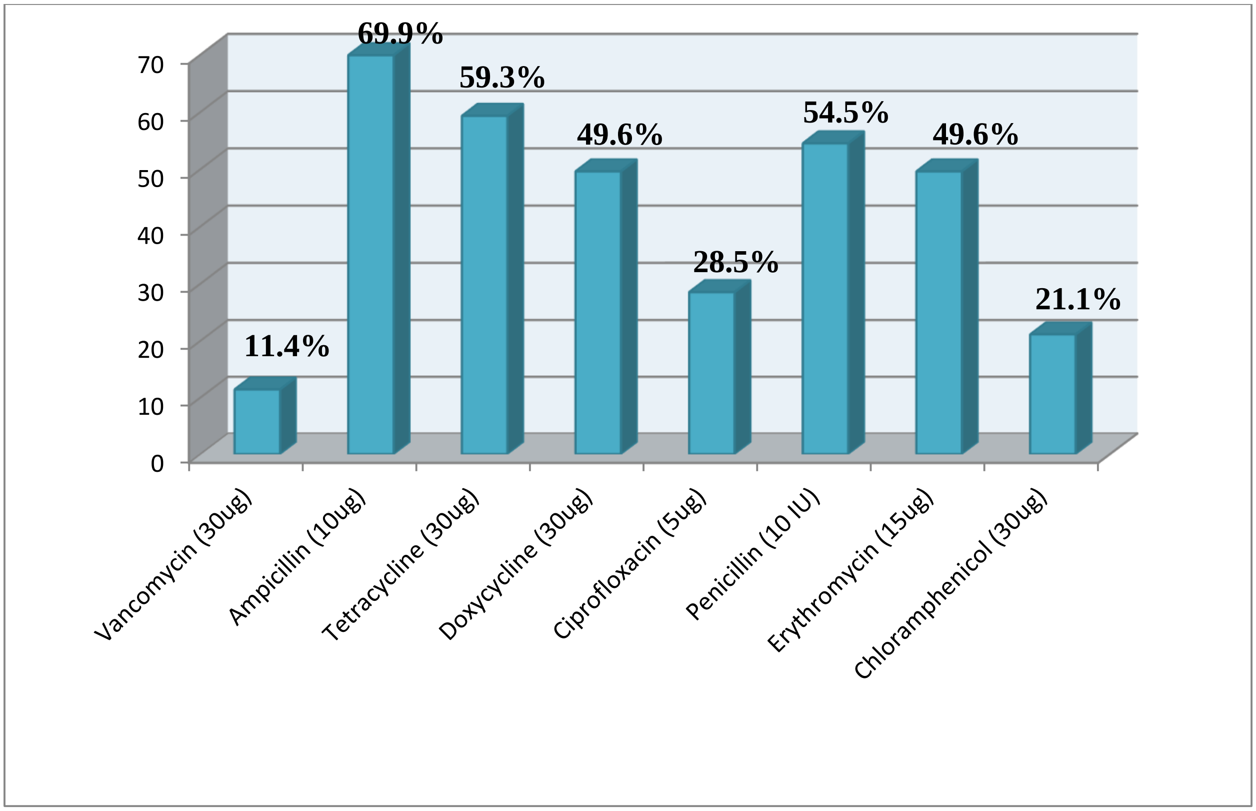

Figure 2. Antimicrobial Resistance Patterns of isolated *Enterococcus* spp among Clients Attending ART Clinic at Arba Minch General Hospital, Southern Ethiopia October 2020 (n=123)

Table 1. Socio-Demographic characteristics of Study Participants Attending ART Clinic at Arba Minch General Hospital, Southern Ethiopia October 2020 (N=200)

**Variables**	**Frequency**	**Percentage (%)**
Sex		
Males	100	50.0
Females	100	50.0
Age		
≤20	12	6.0
21-30	30	15.0
31-40	78	39.0
41-50	47	23.5
≥51	33	16.5
Residence		
Rural	46	23.0
Urban	154	77.0
Educational Status		
Unable to read and write	17	8.5
Able to read and write	42	21.0
Primary (1-8)	62	31.0
Secondary (9-12)	62	31.0
College and above	17	8.5

Table 2. Clinical Characteristics of Study Participants Attending ART Clinic at Arba Minch General Hospital, Southern Ethiopia October 2020 (N=200)

**Visiting Status**	**Frequency**	**Percentage**
Outpatient	187	93.5
Inpatient	13	6.5
CD4 Count		
≤350	32	16.0
>350	168	84.0
Hemoglobin Level		
Low	83	41.5
Normal	111	55.5
High	6	3.0
Previous Antibiotic Treatment		
Never	76	38.0
≥2 weeks	30	15.0
<2 weeks	94	47.0
History of hospitalization in the last six months		
No	174	87.0
Yes	26	13.0
History of previous catheterization		
No	180	90.0
Yes	20	10.0
Comorbid condition (Diabetes)		
No	191	95.5
Yes	9	4.5
Comorbid condition (Renal failure)		
No	193	96.5
Yes	7	3.5

 Click here to access/download;Figure;Table 3.docx

Table 3 P[illegible]ofile of Multidrug Resistance Pattern of VRE isolates among clients Attending ART Clinic at Arba Minch General Hospital, Southern Ethiopia October 2020 (n=14)

**Resistance rate**	**Combination of Antibiotics**	**No. (%) of isolates tested**
R4	G, P, TTC, MAC	1 (7.14)
R5	G, P, TTC, F, MAC	2 (14.28)
R6	G, P, TTC, F, MAC, PH	11 (78.58)

Key; G-glycopeptides (vancomycin), P-penicillins (ampicillin and/or penicillin), TTC-tetracyclines (tetracycline and/or doxycycline), MAC-macrolides (erythromycin), F-fluoroquinolones (ciprofloxacin), PH-phenicols (chloramphenicol), and R4-R6 Number of categories of antibiotics resistance from 4 to 6, respectively

Table 4. Bivariable Logistic Regression Analysis on Factors Affecting Vancomycin Resistant Enterococci among clients Attending ART Clinic at Arba Minch General Hospital, Southern Ethiopia October 2020

Characteristics	Categories	VRE gut colonization		COR	P-value
		**Yes, N (%)**	**No, N (%)**		
Sex	Females	4 (6.8)	55 (93.2)	**0.39**	**0.133***
	Males	10 (15.6)	54 (84.4)	1	
Residence	Urban	11 (11.6)	84 (88.4)	1.09	0.899
	Rural	3 (10.7)	25 (89.3)	1	
Visiting status	Inpatient	3 (30.0)	7 (70.0)	**3.97**	**0.069***
	Outpatient	11 (9.7)	102 (90.3)	1	
CD4 count	≤350	2 (11.8)	15 (88.2)	1.04	0.957
	>350	12 (11.3)	94 (88.7)	1	
Previous Antibiotic Treatment	Never	2 (4.9)	39 (95.1)	1	
	≥2 weeks	8 (40.0)	12 (60.0)	**13.00**	**0.003***
	<2 weeks	4 (6.5)	58 (93.5)	1.34	0.739
History of hospitalization in the last six months	Yes	6 (37.5)	10 (62.5)	**7.43**	**0.002***
	No	8 (7.5)	99 (92.5)	1	
History of previous catheterization	Yes	4 (25.0)	12 (75.0)	**3.23**	**0.078***
	No	10 (9.3)	97 (90.7)	1	
Diabetes Mellitus	Yes	2 (28.6)	5 (71.4)	**3.48**	**0.163***
	No	12 (10.3)	104 (89.7)	1	

*Variables which passed bivariable logistic regression analysis at cut off value <0.25

Table 5. Multivariable Logistic Regression Analysis on Factors Affecting Vancomycin Resistant Enterococci among clients Attending ART Clinic at Arba Minch General Hospital, Southern Ethiopia October 2020

**Characteristics**	**Categories**	**VRE isolates**		**AOR (95%CI)**	**P-value**
		**Yes, N (%)**	**No, N (%)**		
Sex	Females	4 (6.8)	55 (93.2)	0.27 (0.06-1.25)	0.093
	Males	10 (15.6)	54 (84.4)	1	
Visiting status	Inpatient	3 (30.0)	7 (70.0)	1.13 (0.15-8.43)	0.904
	Outpatient	11 (9.7)	102 (90.3)	1	
Previous Antibiotic Treatment	Never	2 (4.9)	39 (95.1)	1	
	≥2 weeks	8 (40.0)	12 (60.0)	**7.35 (1.21-44.64)**	**0.030****
	<2 weeks	4 (6.5)	58 (93.5)	1.08 (0.17-6.80)	0.936
History of hospitalization in the last six months	Yes	6 (37.5)	10 (62.5)	**5.68 (1.09-29.74)**	**0.040****
	No	8 (7.5)	99 (92.5)	1	
History of previous catheterization	Yes	4 (25.0)	12 (75.0)	1.38 (0.27-7.03)	0.701
	No	10 (9.3)	97 (90.7)	1	
Diabetes Mellitus	Yes	2 (28.6)	5 (71.4)	1.81 (0.21 - 15.36)	0.588
	No	12 (10.3)	104 (89.7)	1	
**Significant in multivariable logistic regression at p-value <0.05					

Figure 1

Click here to access/download;Figure;Fig1.JPG

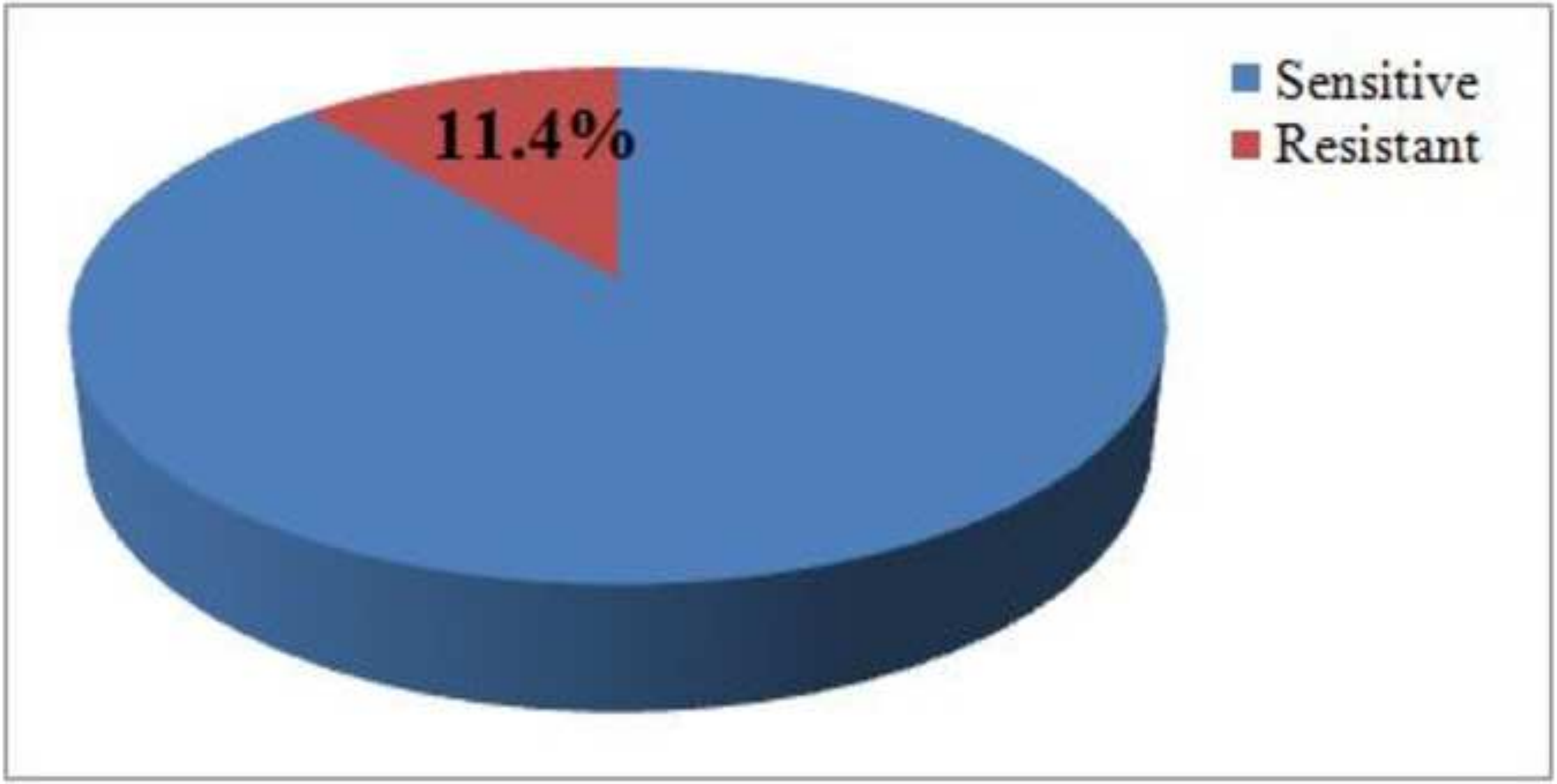

Figure 2

Click here to access/download;Figure;Fig2.JPG

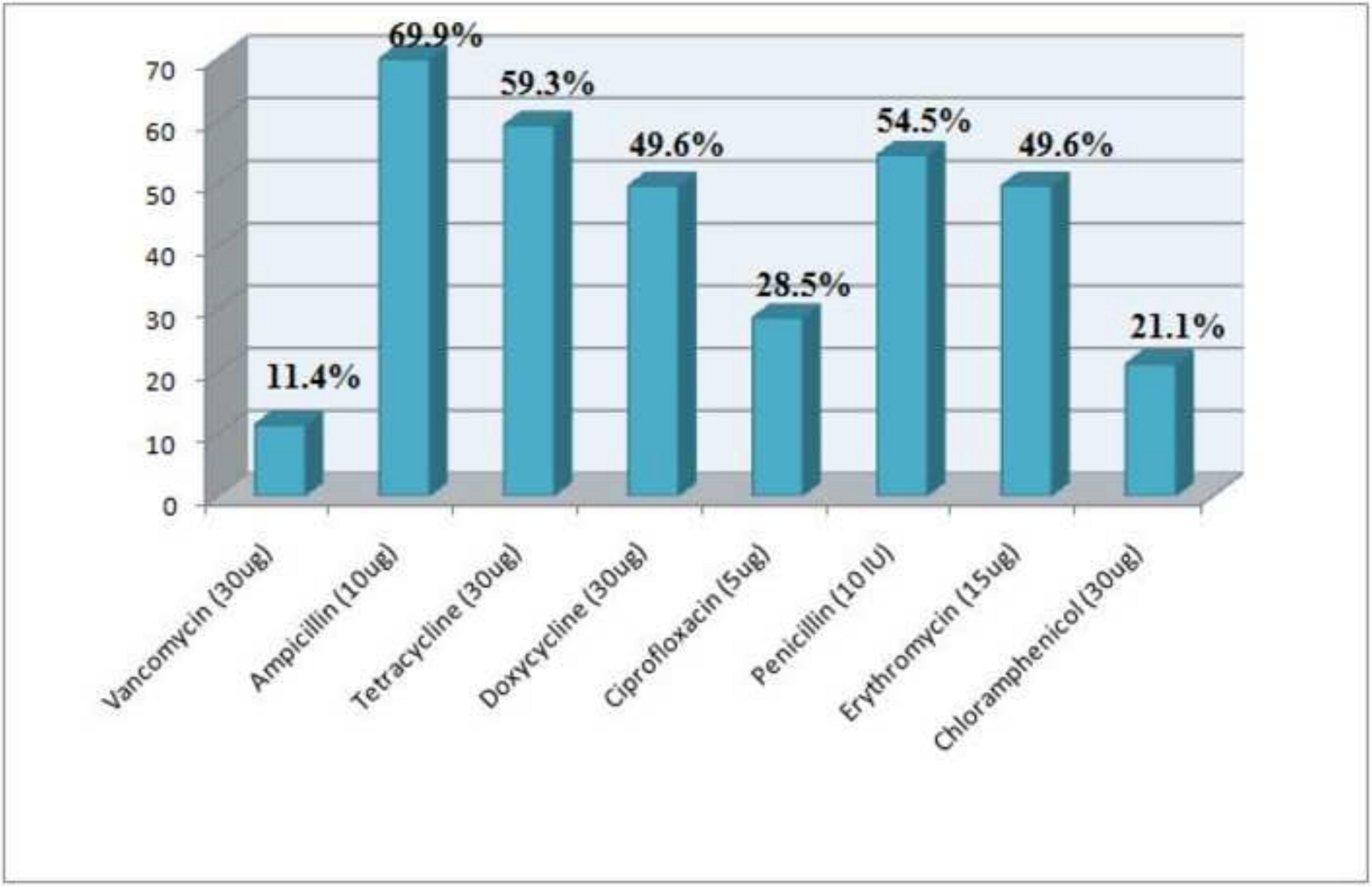

Questionnaire

Click here to access/download
Supporting Information
Questionnaire.docx

Questionnaire

Click here to access/download
Supporting Information
Questionnaire.docx

Vancomycin resistant *Enterococci* and its associated factors among HIV infected patients on anti-Retro viral therapy in Ethiopia

Belayneh Regasa Dadi[1*], Zerihun Solomon[1], Mheret Tesfaye[1]

1. Department of Medical Microbiology, Arba Minch University, Arba Minch, Ethiopia

*Corresponding author
E-mail address of Authors (respectively)
belayjanimen@gmail.com, zerihunsolomon5@gmail.com, mherettesfaye26@gmail.com

Commented [k1]: Italicized

Commented [k2]: As per guideline re-written as
*Corresponding author
E-mail: belayjanimen@gmail.com (BRD)

Abstract

Commented [k3]: Level 1 heading, bold type, font size 18pt

Introduction: The emergence of vancomycin resistant Enterococci (VRE) has alarmed the global infectious diseases community due to its tendency for colonization of the gastrointestinal tract. Human Immunodeficiency Virus (HIV) patients are colonized by vancomycin resistant Enterococci than other groups. The aim of this study was to determine the incidence of vancomycin resistant Enterococci and its associated factors among HIV infected patients on Anti-Retroviral Therapy (ART).

Methodology: Institution based cross sectional study was conducted among HIV infected patients on ART at from June 1 to August 30, 2020. Socio-demographic and clinical data were collected by pre-tested structured questionnaire. Stool sample was collected and processed by standard microbiological techniques. Kirby Bauer Disc diffusion method was used to perform antimicrobial susceptibility testing. Data were entered by Epi data version 4.6.0.2 and analyzed by SPSS version 25. Bivariable and multivariable logistic regression model was used to analyze the association between dependent and independent variables. P-values in the multivariable analysis, adjusted odds ratio (AOR) and 95% confidence interval (CI) were used to determine the strength of association. P-value $\leq$0.05 was considered as significant. P-value $\leq$0.05 was considered as significant.

Results: *Enterococci* spp was isolated on 123/200 (61.50%) respondents. Among these isolates, the incidence of vancomycin resistant Enterococci was 11.4% [95% CI: (6.0-17.0)]. Antimicrobial susceptibility patterns against Enterococci showed highest rate of resistance to ampicillin (69.9%). Multidrug resistances were observed in 49.59% of Enterococci isolates. Study participants who had prior antibioticexposurer more than two weeks [AOR=7.35; 95% CI: (1.2144.64)] and hospitalization for the last six months [AOR=5.68; 95% CI: (1.09 29.74)] were significantly associated with vancomycin resista nt Enterococci.

Conclusions: In our study high incidence of vancomycin resistant Enterococci was found. Previous exposure to antibiotics for more than two weeks and previous hospitalization for more than six months were significant factors for vancomycin resistant Enterococci. The isolated Enterococci had variable degrees of resistance to commonly prescribed antibiotics. Therefore, periodic surveillance on

Commented [k4]: bold

Commented [k5]: Re-written as "The emergence of vancomycin resistant Enterococci (VRE) has alarmed the global community due to its tendency for colonization of the gastrointestinal tract."

Commented [k6]: Re-written as "Methods"

Commented [k7]: These statements were added according to reviewers comment

Commented [k8]: bold

Commented [k9]: bold

antimicrobial resistance pattern, adhering to rational use of antibiotics and implementing infection prevention protocols may reduce colonization by VRE.

Commented [k10]: Re-written as "In our study high incidence of vancomycin resistant *Enterococci* was found. Previous exposure to antibiotics for more than two weeks and hospitalization for more than six months were significantly associated with vancomycin resistant *Enterococci*. The isolated *Enterococci* had variable degrees of resistance to commonly prescribed antibiotics. Therefore, periodic surveillance on antimicrobial resistance pattern, adhering to rational use of antibiotics and implementing infection prevention protocols may reduce colonization by VRE."

Keywords: Human Immunodeficiency Virus, Vancomycin Resistant Enterococci

Introduction

Commented [k11]: Level 1 heading, bold type, font size 18pt

A major problem with the *Enterococci* is that they are very resistant to antibiotics and have ability to survive in harsh environments in community and persist in hospital settings [1]. Because of this, they become important in health facility based settings [2]**.** According to World Health Organization (WHO) report in 2017, vancomycin resistant Enterococci (VRE) is one of the most resistant bacteria in their "Global Priority list of antibiotic-resistant bacteria" [3]. In the same manner, the Center for Disease Control and Prevention (CDC) has classified *Enterococci* among bacteria with a threat level of serious [4].

Currently, VRE are the cause of one-third and one fifth of all health care associated infections in the United States and in some European countries respectively [5]. VRE are well known in causing different enterococcal infections such as infective endocarditis, bacteremia, urinary tract infection, intra-abdominal and pelvic infections, surgical wound infections, and very rarely Central nervous system (CNS) infection [6,7]. Many of these infections originate from intestinal flora of colonized individuals [8].

Enterococci are enteric bacteria that are commonly recovered in feces collected from humans and from a variety of mammals as well as birds, reptiles and insects. The relative importance of *Enterococcus* as a pathogen has increased with the occurrence of high-level resistance to multiple antimicrobial drugs, such as ampicillin and vancomycin [9-13].

Vancomycin is the primary alternative drug to penicillin (plus an aminoglycoside) for treating enterococcal infections [13]. It is active against most Gram positive bacteria and is considered as a drug of 'last resort'. However, nowadays *Enterococcus* spp that are resistant to vancomycin are emerged and spreading all over the world [12].

The term Vancomycin Resistant *Enterococci* (VRE) includes several combinations of enterococcal species including *E. faecium* (77%) and *E. faecalis* (9%), with the remaining 14%

of isolates representing species rarely known in causing serious infections like *E. gallinarum*, *E. casseliflavus*, *E. avium*, and *E. raffinosus* [10,14].

Vancomycin resistant *Enterococci* was first encountered in clinical isolates in England and France in 1986 as *E. faecium*, followed the next year by isolation of Vancomycin Resistant *Enterococcus faecalis* in the United States [15]. They have become an important cause of serious invasive healthcare-associated infections globally [6,7].

The emergence of VRE has alarmed the global infectious diseases community due to its tendency for colonization of the gastrointestinal (GI) tract and transfer of its resistance gene to Methicillin resistant *Staphylococcus aureus* (MRSA) to form a vancomycin-resistant *Staphylococcus aureus* (VRSA) isolate. Besides, VRE have different selection pressures for proliferation and rapid expansion of its resistant populations [16]. Furthermore, few options are left for management of diseases caused by VRE, and hence causing increased mortality on infected individuals [12, 14]. VRE now represent approximately one third of *Enterococcus* isolates [17].

Asymptomatic VRE gut colonization precedes infection with susceptible hosts. Such susceptible hosts are patients who are exposed to multiple and prolonged courses of antimicrobial agents like Human Immunodeficiency virus (HIV) infected individuals, severely ill, hospitalized for long lengths of stay (LOS), living in a long-term care facility, located in close proximity to another colonized or infected patient, or hospitalized in a room previously occupied by a patient colonized with VRE [2,18,19]. Colonization is often obtained by vulnerable hosts in an environment with increased rate of patient colonization with VRE [20].

Vancomycin-resistant enterococci can persist in the environment for elongated periods (>1 week), can contaminate almost any surface, and can be transferred from colonized or infected individual to another by health care workers [9,13]. Transmission of VRE can occur through direct contact with colonized or infected patients, indirect contact via the hands of healthcare workers, via contaminated personal protective equipment (PPE), environmental surfaces or from animals to human through food chain [21,22]. Usually colonization with VRE precedes infection, and the duration of colonization may be extremely extended ranging between 7 weeks and 3 years [22].

Colonization with VRE increases the risk of developing infection up to 5-10 folds [20]. Whether VRE colonization leads to infection depends on the health status of the patient. Whereas

immunocompetent patients colonized with VRE are at low risk for infection, weakened hosts such as HIV infected patients, patients with hematologic disorders, transplant recipients, or severely ill patients have an increased likelihood of developing infection following colonization [19,20].

Compromised immune system of HIV infected patients increases the chances of acquiring various opportunistic infections [23]. These individuals are at increased risk of developing infections, including infections caused by resistant bacteria pathogens [24]. VRE is an important opportunistic bacterial pathogen causing significant morbidity and mortality in immunocompromised individuals like HIV patients [8,25].

The prevalence of VRE was reported in Europe, Asia, Australia, South America and some African countries [1,5,24,26]. However, there is no sufficient data available on the prevalence and risk factors of VRE in developing countries like Ethiopia. Therefore, this study was conducted with the aim of determining the prevalence of vancomycin resistant enterococci gut colonization and its associated factors among HIV infected patients on Anti-Retroviral Therapy (ART).

Methodology

Study design, period and setting

The study was conducted in Arba Minch General Hospital from June 1 to August 30, 2020. The hospital is located in Arba Minch town Gamo zone, 505 km South of Addis Ababa. The hospital has ART clinic where it provides different services for HIV- infected patients. The total population of Arba Minch town from 2007 central statistical agency (CSA) census report was 74,879, of whom 39,208 were men and 35,671 women [27]. The inclusion criteria was all HIV infected patients on ART at Arba Minch General Hospital, ART clinic during the study period. Exclusion criteria are those HIV infected patients who don't have Parent or guardian assent if they are <18 years old and those HIV infected patients who were critically ill and unable to respond.

Commented [k12]: According to Reviewers comment Introduction section is re-written as "A major problem with the *Enterococci* is that they are very resistant to antibiotics and have ability to survive in harsh environments in community and persist in hospital settings [1]. Because of this, they become important in health facility based settings [2]. According to World Health Organization (WHO) report in 2017, vancomycin resistant Enterococci (VRE) is one of the most resistant bacteria in their "Global Priority list of antibiotic-resistant bacteria" [3]. In the same manner, the Center for Disease Control and Prevention (CDC) has classified *Enterococci* among bacteria with a threat level of serious [4]. Currently, VRE are the cause of one-third and one fifth of all health care associated infections in the United States and in some European countries respectively [5].
VRE are known in causing different enterococcal infections such as infective endocarditis, bacteremia, urinary tract infection, intra-abdominal and pelvic infections, surgical wound infections, and very rarely Central nervous system (CNS) infection. Many of these infections originate from intestinal flora of colonized individuals. VRE have different selection pressures for proliferation and rapid expansion of its resistant populations. Furthermore, few options are left for management of diseases caused by VRE, and hence causing increased mortality on infected individuals. VRE now represent approximately one third of *Enterococcus* isolates [6-17].
Asymptomatic VRE gut colonization precedes infection with susceptible hosts. Such susceptible hosts are patients who are exposed to multiple and prolonged courses of antimicrobial agents like Human Immunodeficiency virus (HIV) infected individuals, severely ill, hospitalized for long lengths of stay (LOS), living in a long-term care facility, located in close proximity to another colonized or infected patient, or hospitalized in a room previously occupied by a patient colonized with VRE. Colonization is often obtained by vulnerable hosts in an environment with increased rate of patient colonization with VRE [2,18-26]. The prevalence of VRE was reported in Europe, Asia, Australia, South America and some African countries [1,5,24,26]. However, there is no sufficient data available on the prevalence and risk factors of VRE in developing countries like Ethiopia. Therefore, this study was conducted with the aim of determining the prevalence of vancomycin resistant enterococci and its associated factors among HIV infected patients on Anti-Retroviral Therapy (ART).
"

Commented [k13]: Name changed from "Methodology" to "Materials and Methods" Level 1 heading, bold type, font size 18pt

Commented [k14]: Level 2 headings for subsections, bold, font size 16pt

Commented [k15]: Re-written as "The study was conducted in Arba Minch General Hospital (Arba Minch, Ethiopia) from June 1 to August 30, 2020. The hospital has ART clinic where it provides different services for HIV- infected patients. The total population of Arba Minch town from 2007 central statistical agency (CSA) census report was 74,879, of whom 39,208 were men and 35,671 women [27]. The inclusion criteria was all HIV infected patients on ART during the study period. Exclusion criteria were HIV infected patients who don't have Parent or guardian assent if they are <18 years old and those HIV infected patients who were critically ill and unable to respond."

Sampling technique

Commented [k16]: Level 2 headings for subsections, bold, font size 16pt

Systematic random sampling technique was used to select the study participants. Based on the 2020 three months (June-August, 2020) data obtained during COVID-19 pandemic from Arba Minch General Hospital ART clinic, N=366 (N= total number of study participants) HIV infected patients has visited the ART clinic. Assuming the same number of HIV- infected patients for the study period (June-August, 2020) and taking sample size of 200, the kth value (k=N/n=366/2002) is found to be 2. The first one is selected with lottery method from 1st and 2nd patients and found to be 2nd patient. Then every 2nd patient was included.

Dependent variable was VRE gut colonization and independent variables were: Age, Sex, CD4 count, Level of hemoglobin, Previous antibiotic treatment for >2 weeks, Current visiting status (Inpatient or Outpatient), History of hospitalization in the last six months, Urinary catheterization, Co-morbid conditions (diabetes and renal failure).

Data collection and laboratory processing

Commented [k17]: Level 2 headings for subsections, bold, font size 16pt

A pretested well designed structured questionnaire was used to collect data from the study participants. The questionnaire was designed in English and translated in to Amharic language. The tool had two sections and was adapted by reviewing different literatures. The first section consisted of socio demographic questions and the second section embraced clinical data.

Commented [k18]: Re-written as "A pretested well designed structured questionnaire was used to collect data from the study participants. The questionnaire was designed by reviewing different literatures and prepared in English and Amharic languages."

Upon the arrival of each study participant at ART clinic, written assent/consent was obtained. Socio-demographic and clinical data were collected by two nurses using a pretested well designed structured questionnaire through face-to-face interview. Moreover, recent CD4 count and hemoglobin level of respondents have been taken from ART clinic logbook.

Isolation and Identification of *Enterococci*

Commented [k19]: Level 2 headings for subsections, bold, font size 16pt

Each patient was instructed how to collect stool specimens, and informed to collect about 2gm of the faecal specimen in a sterile wide-mouth screw capped container labelled with the unique sample number, date, and time of collection. The collected stool specimens were transported to Arba Minch University College of Medicine and Health Sciences Microbiology and Parasitology laboratory.

Commented [k20]: Re-written as "Patients were instructed to collect about 2gm of the faecal specimen in a sterile wide-mouth screw capped container labeled with the unique sample number, date, and time of collection. The collected stool specimens were transported to Arba Minch University, College of Medicine and Health Sciences, Microbiology and Parasitology laboratory."

Immediately after delivery, the transported stool specimens was streaked on Bile Esculin Azide agar (BEAA) (Park Scientific Limited, 24 Low Farm Place, Moulton Park, Northampton, NN3 6HY) and incubated for 24 hours at 37°C. Plates were observed for appearance of characteristic growth with brown-black colored colonies in the medium and dark halo centers. Typical characteristic colonies were selected randomly for characterization and presumptive identification of *Enterococci* by Gram stains, Catalase test, Salt tolerance test and Heat tolerance test [29] (Figure 2).

Commented [k21]: Deleted (inappropriately placed)

Antimicrobial Susceptibility Testing

Commented [k22]: Level 2 headings for subsections, bold, font size 16pt

Commented [k23]: This sentence is deleted based on comment given by reviewers

The antimicrobial susceptibility testing was performed using Kirby Bauer disc diffusion method according to Clinical Laboratory Standards Institute guidelines (CLSI). After a pure colony was obtained, a loop full of bacteria were taken, transferred to a tube containing 5 ml of sterile normal saline (0.85% NaCl) and mixed gently until it formed a homogenous suspension.

The turbidity of the suspension was determined by comparison with 0.5 McFarland standards. A sterile swab was dipped into the suspension, and excess suspension was removed by pressing the swab against the wall of the tube. The entire surface of Muller Hinton Agar plate was uniformly flooded with suspensions and allowed to dry for about 3-5 minutes.

The antimicrobial impregnated disks were placed by using sterile forceps at least 24 mm away from each other to avoid the overlapping zone of inhibition. The disks were placed on agar plates and allowed to stand for 30 minutes to dissolve the antibiotics in the media [16]. The plates were then inverted and incubated at 37°c for 16-18 hours and inhibition zone was measured using a ruler.

For instance, in the case of vancomycin the plates are inverted and incubated at 37°c, and were held a full 16-18 hours for accurate detection of resistance. Zones were examined using transmitted light; the presence of a haze or any growth within the zone of inhibition indicates resistance for vancomycin. Grades of susceptibility pattern were recognized as sensitive, intermediate and resistant by comparison of zone of inhibition according to the 2018 CLSI guidelines [30].

Antimicrobial susceptibility patterns of *Enterococci* were assessed against the following antibiotic discs: Penicillin (10 IU), Ampicillin (10 µg), Tetracycline (30µg), Doxycycline (30µg), Ciprofloxacin (5µg), Vancomycin (30 µg), Erythromycin (15µg) and Chloramphenicol (30µg), and were interpreted according to CLSI M100- 2018 (30). Antibiotics were selected based on CLSI recommendation, local availability (in health facility) and feasibility (cost and method of antimicrobial susceptibility test).

Data Quality Assurance

The reliability of the study findings were guaranteed through performing a pretest of 5 % (n=10) on HIV infected individuals at Arba Minch Town Shecha Health Center, and implementing the standard quality measures through the entire process of data collection and laboratory work. Accordingly, one day training was given for data and sample collectors concerning on the research objective, data collection tools, sample collection procedures, and infection prevention protocols to be taken related with COVID-19 Pandemic.

The data were checked for completeness, accuracy, clarity, and consistency by the principal investigator on a daily basis. Standard Operating Procedures (SOP) for each procedure were strictly followed. It was verified that the media and reagents met expiration date and quality control parameters per CLSI guideline. All culture media were prepared following the manufacturer's instruction and sterility of the culture media was tested by incubating 5% of the batch at 35–37°C overnight for evaluation of possible contamination.

Moreover, *E. faecalis* ATCC 29212 and *Staphylococcus aureus* ATCC 25923 standard control strains obtained from Ethiopian Public Health Institution (EPHI) were used as a quality control for biochemical tests and agar plates including Mueller–Hinton agar with vancomycin disc to ensure testing performance of the potency of the disc as per CLSI 2018 guidelines [30].

Statistical analysis

Data were checked for its completeness, entered by Epi data version 4.6.0.2 software, and analyzed by SPSS version 25. Descriptive statistics like frequency, mean and percentage were used. The fitness of the model was checked by Hosmer-Lemeshow goodness of fit test.

Commented [k24]: Re-written as "The antimicrobial susceptibility testing was performed using Kirby Bauer disc diffusion method according to Clinical Laboratory Standards Institute guidelines (CLSI) [30]. After a pure colony was obtained, a loop full of bacteria were taken, transferred to a tube containing 5 ml of sterile normal saline (0.85% NaCl) and mixed gently until it formed a homogenous suspension. The turbidity of the suspension was determined by comparison with 0.5 McFarland standards. A sterile swab was dipped into the suspension, and excess suspension was removed by pressing the swab against the wall of the tube. The entire surface of Muller Hinton Agar plate was uniformly flooded with suspensions and allowed to dry for about 3-5 minutes. The antimicrobial impregnated disks were placed by using sterile forceps at least 24 mm away from each other to avoid the overlapping zone of inhibition. The disks were placed on agar plates and allowed to stand for 30 minutes to dissolve the antibiotics in the media [16]. The plates were then inverted and incubated at 37ºc for 16-18 hours and inhibition zone was measured using a ruler.

In the case of vancomycin, the plates were inverted and incubated at 37ºc, and held a full 16-18 hours for accurate detection of resistance. Zones were examined using transmitted light; the presence of a haze or any growth within the zone of inhibition indicates resistance for vancomycin. Grades of susceptibility pattern were recognized as sensitive, intermediate and resistant by comparison of zone of inhibition according to the 2018 CLSI guidelines [30]. Antimicrobial susceptibility patterns of *Enterococci* were also assessed against the following antibiotic discs: Penicillin (10 IU), Ampicillin (10 µg), Tetracycline (30µg), Doxycycline (30µg), Ciprofloxacin (5µg), Vancomycin (30 µg), Erythromycin (15µg) and Chloramphenicol (30µg). Interpretations of results were made according to CLSI (30). Antibiotics were selected based on CLSI recommendation, local availability (in health facility) and feasibility (cost and method of antimicrobial susceptibility test)."

Commented [k25]: Level 2 headings for subsections, bold, font size 16pt

Commented [k26]: Re-written as "A pretest was done on 5 % (n=10) HIV infected individuals. Quality control measures were implementing throughout the entire process of data collection and laboratory work. Training was given for data and sample collectors concerning on the research objective, data collection tools, sample collection procedures, and infection prevention protocols to be taken related with COVID-19 Pandemic. Standard Operating Procedures (SOP) were prepared and implemented strictly. All culture media were prepared following the manufacturer's instruction and sterility of the culture media was tested by incubating 5% of the batch at 35–37°C overnight for evaluation of possible contamination. Standard control strains *E. faecalis* ATCC 29212 and *Staphylococcus aureus* ATCC 25923 were used as a positive control."

Commented [k27]: Level 2 headings for subsections, bold, font size 16pt

Bivariable and multivariable logistic regression model was used to analyze the association between dependent and independent variables. Those variables with P-value < 0.25 in bivariate analysis were considered as candidate for further multivariable analysis. P-values in the multivariable analysis, adjusted odds ratio (AOR) and 95% confidence interval (CI) were used to determine the strength of association. P-value <0.05 was considered as statistically significant. Finally, the study findings were explained in words, figures and tables.

Ethics approval and consent to participate

Ethical clearance was obtained from Institutional Review Board (IRB) of Arba Minch University, College of Medicine and Health Sciences, and letter of permission to conduct the study was written to Arba Minch General Hospital. Official permission from Arba Minch General Hospital and an informed written assent/consent from each study participants (assent of those <18 age group from their family or guardian) were obtained.

All information that identifies study subjects were given code numbers and were kept confidential. The purpose of the study was clearly described to the study participants and the specimens collected from the patients were analyzed for the intended purpose only. For each confirmed resistant case, the responsible clinicians of the patient were informed for appropriate management.

Results

Socio-demographic characteristics of study participants

A total of 200 study participants were enrolled in this study. Among these the ratio of male to female was 1:1. The mean age and standard deviation was 38.68 ± 10.97 ranging from 18-67 years. More than three fourth of the study participants 154 (77.0%) came from urban settings (**Table 1**).

Table 1. Socio-demographic characteristics of study participants attending ART Clinic at Arba Minch General Hospital, Southern Ethiopia October 2020 (N=200)

**Variables**	**Frequency**	**Percentage (%)**

Commented [k28]: Re-written as "Data were checked for its completeness, entered by Epi data version 4.6.0.2 software, and analyzed by SPSS version 25. The fitness of the model was checked by Hosmer-Lemeshow goodness of fit test. Bivariable and multivariable logistic regression model was used to analyze the association between dependent and independent variables. Those variables with P-value < 0.25 in bivariate analysis were considered as candidate for further multivariable analysis. P-values in the multivariable analysis, adjusted odds ratio (AOR) and 95% confidence interval (CI) were used to determine the strength of association. P-value <0.05 was considered as statistically significant."

Commented [k29]: This section is moved here (Methods section) as per the comment given by editor

Commented [k30]: Level 1 heading, bold type, font size 18

Commented [k31]: Level 2 headings for subsections, bold, font size 16pt

Commented [k32]: bold

Commented [k33]: Table 1 included in the manuscript

Sex		
Males	100	50.0
Females	100	50.0
**Age**		
≤20	12	6.0
21-30	30	15.0
31-40	78	39.0
41-50	47	23.5
≥51	33	16.5
**Residence**		
Rural	46	23.0
Urban	154	77.0
**Educational Status**		
Unable to read and write	17	8.5
Able to read and write	42	21.0
Primary (1-8)	62	31.0
Secondary (9-12)	62	31.0
College and above	17	8.5

Clinical characteristics of study participants

Commented [k34]: Level 2 headings for subsections, bold, font size 16pt

The clinical data showed that majority of the participants 187 (93.5%) were outpatients and 168 (84.0%) of the participants had CD4 count >350. Less than half of the respondents 83 (41.5%) had low hemoglobin level and 94 (47.0%) had history of previous antibiotic use for <2 weeks. Majority of the study participants 174 (87.0%) didn't have previous history of hospitalization in the last six months and 180 (90.0%) didn't have history of previous catheterization. Likewise, majority of the respondents 191 (95.5%) and 193 (96.5%) didn't have Diabetes Mellitus and Renal failure respectively (**Table 2**).

Commented [k35]: bold

Table 2. Clinical Characteristics of Study Participants Attending ART Clinic at Arba Minch General Hospital, Southern Ethiopia October 2020 (N=200)

Commented [k36]: Table 2 included in the manuscript

Visiting Status	Frequency	Percentage

Outpatient	187	93.5
Inpatient	13	6.5
**CD_4+ Count**		
≤350	32	16.0
>350	168	84.0
**Hemoglobin Level**		
Low	83	41.5
Normal	111	55.5
High	6	3.0
**Previous Antibiotic Treatment**		
Never	76	38.0
≥2 weeks	30	15.0
<2 weeks	94	47.0
**History of hospitalization in the last six months**		
No	174	87.0
Yes	26	13.0
**History of previous catheterization**		
No	180	90.0
Yes	20	10.0
**Comorbid condition (Diabetes)**		
No	191	95.5
Yes	9	4.5
**Comorbid condition (Renal failure)**		
No	193	96.5
Yes	7	3.5

Incidence of vancomycin resistant *Enterococcus*

From the total of 200 study participants, *Enterococci* species was isolated from 123 (61.50%) patients. Among these isolates, 14 (11.4%) were vancomycin resistant (95% CI: 6.0-17.0%) (**Figure 1**).

Commented [k37]: Level 2 headings for subsections, bold, font size 16pt

Commented [k38]: bold

Figure 1. Incidence of vancomycin resistant *Enterococci* among clients attending ART Clinic at Arba Minch General Hospital, Southern Ethiopia October 2020 (N=123)

Commented [k39]: This sentence is added as per the guideline

Antimicrobial resistance profile of isolated *Enterococci*

Commented [k40]: Level 2 headings for subsections, bold, font size 16pt

Among 123 *Enterococci* isolates tested for commonly prescribed antimicrobial agents the highest rate of resistance was observed against ampicillin in which more than two thirds 86 (69.9%) of the isolates were resistant (**Figure 2**).

Commented [k41]: bold

Figure 2. Antimicrobial resistance patterns of isolated *Enterococcus* spp among clients attending ART Clinic at Arba Minch General Hospital, Southern Ethiopia October 2020 (n=123)

Commented [k42]: This sentence is added as per the guideline

Multidrug resistances (MDR) were observed in slightly less than half 61 (49.59%) of *Enterococci* isolates and less than one tenth 11 (8.94%) of the isolates were resistant to all antimicrobials tested. All VRE isolates were MDR (**Table 3**).

Commented [k43]: bold

Table 3. Profile of Multidrug Resistance Pattern of VRE isolates among clients Attending ART Clinic at Arba Minch General Hospital, Southern Ethiopia October 2020 (n=14)

**Resistance rate**	**Combination of Antibiotics**	**No. (%) of isolates tested**
R4	G, P, TTC, MAC	1 (7.14)
R5	G, P, TTC, F, MAC	2 (14.28)
R6	G, P, TTC, F, MAC, PH	11 (78.58)

Key; G-glycopeptides (vancomycin), P-penicillins (ampicillin and/or penicillin), TTC-tetracyclines (tetracycline and/or doxycycline), MAC-macrolides (erythromycin), F-fluoroquinolones (ciprofloxacin), PH-phenicols (chloramphenicol), and R4-R6 Number of categories of antibiotics resistance from 4 to 6, respectively

Commented [k44]: Table 3 included in the manuscript

Factors associated with vancomycin resistant *Enterococcus* gut colonization

Commented [k45]: Level 2 headings for subsections, bold, font size 16pt

Factors independently associated with VRE gut colonization on bivariable analysis, and fit into a multivariable logistic regression model were sex, visiting status, history of catheterization,

previous antibiotics treatment, history of hospitalization in the last six months and presence of Diabetes Mellitus (**Table 4 and 5**).

Commented [k46]: bold

Table 4. Bivariable Logistic Regression analysis on factors affecting vancomycin resistant *Enterococci* among clients attending ART Clinic at Arba Minch General Hospital, Southern Ethiopia October 2020

Commented [k47]: Table 4 and 5 included in the manuscript

**Characteristics**	**Categories**	**VRE gut colonization**		**COR**	**P-value**
		**Yes, N (%)**	**No, N (%)**		
Sex	Females	4 (6.8)	55 (93.2)	**0.39**	**0.133***
	Males	10 (15.6)	54 (84.4)	1	
Residence	Urban	11 (11.6)	84 (88.4)	1.09	0.899
	Rural	3 (10.7)	25 (89.3)	1	
Visiting status	Inpatient	3 (30.0)	7 (70.0)	**3.97**	**0.069***
	Outpatient	11 (9.7)	102 (90.3)	1	
CD4 count	≤350	2 (11.8)	15 (88.2)	1.04	0.957
	>350	12 (11.3)	94 (88.7)	1	
Previous Antibiotic Treatment	Never	2 (4.9)	39 (95.1)	1	
	≥2 weeks	8 (40.0)	12 (60.0)	**13.00**	**0.003***
	<2 weeks	4 (6.5)	58 (93.5)	1.34	0.739
History of hospitalization in the last six months	Yes	6 (37.5)	10 (62.5)	**7.43**	**0.002***
	No	8 (7.5)	99 (92.5)	1	
History of previous catheterization	Yes	4 (25.0)	12 (75.0)	**3.23**	**0.078***
	No	10 (9.3)	97 (90.7)	1	
Diabetes Mellitus	Yes	2 (28.6)	5 (71.4)	**3.48**	**0.163***
	No	12 (10.3)	104 (89.7)	1	
*Variables which passed bivariable logistic regression analysis at cut off value <0.25					

Table 5. Multivariable Logistic Regression analysis on factors affecting vancomycin resistant *Enterococci* among clients attending ART Clinic at Arba Minch General Hospital, Southern Ethiopia October 2020

Characteristics	Categories	VRE isolates		AOR (95%CI)	P-value
		Yes, N (%)	No, N (%)		
Sex	Females	4 (6.8)	55 (93.2)	0.27 (0.06-1.25)	0.093
	Males	10 (15.6)	54 (84.4)	1	
Visiting status	Inpatient	3 (30.0)	7 (70.0)	1.13 (0.15-8.43)	0.904
	Outpatient	11 (9.7)	102 (90.3)	1	
Previous Antibiotic Treatment	Never	2 (4.9)	39 (95.1)	1	
	≥2 weeks	8 (40.0)	12 (60.0)	**7.35 (1.21-44.64)**	**0.030****
	<2 weeks	4 (6.5)	58 (93.5)	1.08 (0.17-6.80)	0.936
History of hospitalization in the last six months	Yes	6 (37.5)	10 (62.5)	**5.68 (1.09-29.74)**	**0.040****
	No	8 (7.5)	99 (92.5)	1	
History of previous catheterization	Yes	4 (25.0)	12 (75.0)	1.38 (0.27-7.03)	0.701
	No	10 (9.3)	97 (90.7)	1	
Diabetes Mellitus	Yes	2 (28.6)	5 (71.4)	1.81 (0.21 - 15.36)	0.588
	No	12 (10.3)	104 (89.7)	1	
**Significant in multivariable logistic regression at p-value <0.05					

Discussion

Commented [k48]: Level 1 heading, bold type, font size 18

The hasty emergence and the increasing incidence of colonization with VRE have become challenging healthcare problems that have caused serious concern to health care providers and health authorities [2]. In the present study, the prevalence of VRE among HIV infected patients was 11.4% (95% CI: 6.0-17.0%). This prevalence rate was consistent with other studies conducted in South Africa (14.5%) [21], West Amhara, Ethiopia (7.7%) [2], Addis Ababa, Ethiopia (6.7%) [31] and Gondar, Northwest Ethiopia (7.8%) [18].

However, the prevalence of VRE in our study was lower than the report from Ireland (44.1%) [4], Germany (26.1%) [32] Brazil (23.4%) [1], India (19.6%) [33], Saud Arabia (17.3%) [5] and Iraq (46.4%) [17]. The lower prevalence in the present study might be due to the variation in study settings in which most of the previous countries have been using oral and intravenous vancomycin massively for human diseases [34,35], and variation in study participants in which in the previous studies most of the study participants were hospitalized patients and critically ill patients in ICU who were frequently exposed to different antibiotics and experienced insertion of external devices like catheter [7].

On the other hand, this prevalence is higher than the report from USA (4.7%) [36], Nigeria (4.07%) [37] and Ethiopia (5.9%) [16]. The gradual increase in the prevalence of VRE might have contributed to this higher prevalence as supported by other studies [32,38].

Enterococci isolates in our study showed various resistances to the tested antibiotics; namely, 69.9% to ampicillin, 54.5% to penicillin, 49.6% to erythromycin, 59.3% to tetracycline, 28.5% to ciprofloxacin and 21.1% to chloramphenicol. This finding is in line with studies conducted in India 64.9% [33] and Ethiopia 66.7% [39] for ampicillin; Ethiopia 64.9% [40], 68% [39] for tetracycline; and Brazil (45.7%) [1] and Ethiopia 42.7% [9] (53.3%) [31] for erythromycin.

However, the resistance profile in our study is lower than previous studies in India for tested antibiotics 75.9% for penicillin, 84.5% for tetracycline, 95.5% for ciprofloxacin, 92.1% for erythromycin and 42.3% for chloramphenicol [33]; Uganda 69.4% [15] and Ethiopia 77.3% [41] for tetracycline; and Iraq (85.7%) [17], Uganda (72%) [15] and Ethiopia 63.2% [41] erythromycin; This lower resistance pattern might be due to variation in study participants in which most of the participants in the previous studies were hospitalized patients who were taking different antibiotics that might be contributed for emergence of high rate of drug resistant microorganisms including *Enterococci*.

On the other hand, the antibiotic resistance profile in our study is higher as compared to studies conducted in Iran 41.2% [42], Brazil 0% [1], Uganda 1.4% [15] and Ethiopia 36% [41] for ampicillin; Brazil 32.6% [1] and Ethiopia 37.7% [2] for tetracycline; and Brazil 10.9% [1] for chloramphenicol. This discrepancy might be due to the fact that we are in the time of gradual increase in antibiotic resistance pattern which in turn might be due to overuse or misuse of antibiotics, inappropriate antibiotics prescription, extensive antibiotics use for agricultural purpose, mutation, gene transfer among bacteria, etc. [4].

Commented [k49]: Re-written as "variation in study participants where the previous studies participants were hospitalized patients and critically ill patients in ICU who were frequently exposed to different antibiotics and experienced insertion of external devices like catheter [7]."

Commented [k50]: Re-written as "On the other hand, the prevalence in our study was higher than studies conducted in USA (4.7%) [36], Nigeria (4.07%) [37] and Ethiopia (5.9%) [16]. The gradual increase and clonal expansion in the prevalence of VRE might have contributed to this higher prevalence [32,38]."

Commented [k51]: Re-written as "*Enterococci* isolates showed various resistances to the tested antibiotics; namely, 69.9% to ampicillin, 54.5% to penicillin, 49.6% to erythromycin, 59.3% to tetracycline, 28.5% to ciprofloxacin and 21.1% to chloramphenicol. These findings are comparable with studies conducted in India 64.9% [33] and Ethiopia 66.7% [39] for ampicillin; Ethiopia 64.9% [40], 68% [39] for tetracycline; and Brazil 45.7% [1] and Ethiopia 42.7% [9] 53.3% [31] for erythromycin. However, the resistance profiles in our study is lower than previous studies in India for tested antibiotics 75.9% for penicillin, 84.5% for tetracycline, 95.5% for ciprofloxacin, 92.1% for erythromycin and 42.3% for chloramphenicol [33]; Uganda 69.4% [15] and Ethiopia 77.3% [41] for tetracycline; and Iraq (85.7%) [17], Uganda (72%) [15] and Ethiopia 63.2% [41] erythromycin; These lower drug resistance patterns might be due to variations in sample size, methodology and study participants where most of the participants in the previous studies were hospitalized patients who were taking different antibiotics that might have been contributed for emergence of high rate of drug resistance."

Commented [k52]: Re-written as "On the other hand, the antibiotic resistance profile in our study is higher than studies conducted in Iran 41.2% [42], Brazil 0% [1], Uganda 1.4% [15] and Ethiopia 36% [41] for ampicillin; Brazil 32.6% [1] and Ethiopia 37.7% [2] for tetracycline; and Brazil 10.9% [1] for chloramphenicol. The high drug resistance patterns might be due to overuse or misuse of antibiotics and inappropriate antibiotics prescription which is very common practice in Ethiopia. Additional reasons for high drug resistance might be extensive antibiotics use of antibiotics for agricultural purpose, mutation and gene transfer among bacteria [4]. "

Moreover, the present study also showed that 49.6% of *Enterococci* isolates were multidrug resistant. This result is lower than the findings in Iraq (85.7%) [17] and Ethiopia 80.8% [41]. In contrary, the result is higher than the finding in Ethiopia 29.5% [16]. The discrepancy of the result might be due to variation in geographical distribution of strain, trend and frequency of antibiotic prescription, community drug usage practice.

Regarding associated factors, the current study revealed that HIV patients who were exposed to antibiotics for more than two weeks previously were seven times more likely to be colonized with VRE as compared with HIV patients who never exposed to antibiotics previously [AOR=7.35; 95% CI: (1.21-44.64); P- value= 0.030]. This result is in agreement with other studies conducted in Brazil [1], South Korea [43], Egypt [44], and Ethiopia [2]. The reason might be a prior exposure to antibiotics for a prolonged duration can cause VRE colonization due to the fact that the antibiotics exert selective pressure to *Enterococci* and alter the competing microbial flora in the GI tract allowing VRE to predominate as evidenced by other studies [7,34].

Moreover, the present study also showed that HIV patients who had history of hospitalization in the last six months were six times more likely to be colonized with VRE as compared with HIV patients who didn't have history of hospitalization in the last six months [AOR=5.68; 95% CI: (1.09-29.74); P-value = 0.040]. The finding is consistent with previous studies done in USA [36], South Korea [43] and Ethiopia [2]. The reason might be VRE have been isolated from virtually every object within patient rooms since they are intrinsically resistant to several commonly used antibiotics in hospital and have ability to acquire resistance genes. Besides, they are ubiquitous in their presence and have high survivability on dry surfaces, thereby causing high VRE transmission rates within healthcare facilities [7,9].

Limitations of the study

The isolated *Enterococci* were not identified to species level and no molecular characterization done due to resource limitation.

Commented [k53]: Re-written as "Our study showed that HIV patients who were previously exposed to antibiotics for more than two weeks were seven times more likely to be colonized with VRE as compared with HIV patients who never exposed to antibiotics previously [AOR=7.35; 95% CI: (1.21-44.64); P- value= 0.030]. This result is in agreement with other studies conducted in Brazil [1], South Korea [43], Egypt [44], and Ethiopia [2]. The reason might be a prior exposure to antibiotics for a prolonged duration can cause VRE colonization due to the fact that the antibiotics exert selective pressure to *Enterococci* and alter the competing microbial flora in the GI tract allowing VRE to predominate as evidenced by other studies [7,34]."

Commented [k54]: Re-written as "The present study showed that HIV patients who had history of hospitalization for the last six months were six times more likely to be colonized with VRE as compared with HIV patients who didn't have history of hospitalization for the last six months [AOR=5.68; 95% CI: (1.09-29.74); P-value = 0.040]. The finding is consistent with previous studies done in USA [36], South Korea [43] and Ethiopia [2]. The reason might be VRE have been isolated from virtually every object within patient rooms since they are intrinsically resistant to several commonly used antibiotics in hospital and have ability to acquire resistance genes. Besides, they are ubiquitous in their presence and have high survivability on dry surfaces, thereby causing high VRE transmission rates within healthcare facilities [7,9]."

Commented [k55]: Level 1 heading, bold type, font size 18

Commented [k56]: Based on the comments given by the Reviewers re-written as "The isolated *Enterococci* were not identified to species level; molecular characterization and MIC were done due to resource limitation and budget constraints."

Conclusions

Commented [k57]: Level 1 heading, bold type, font size 18

In our study high incidence of vancomycin resistant Enterococci was found. Previous exposure to antibiotics for more than two weeks and previous hospitalization for more than six months were significant factors for vancomycin resistant enterococci gut colonization. The study also showed that the isolated *Enterococci* had variable degrees of resistance to commonly prescribed antibiotics.

Therefore periodic surveillance on antimicrobial resistance pattern of *Enterococcus* species is important for proper treatment, health professionals should strictly follow infection prevention protocols and further studies should be conducted on species identification and molecular characterization of *Enterococci*.

List of abbreviations

Commented [k58]: Level 1 heading, bold type, font size 18

ART: Anti-Retroviral Therapy **AOR**: Adjusted odd ratio **BEAA**: Bile Esculin Azide agar **CDC**: Center for Disease Control **CI**: Confidence Interval **CLSI**: Clinical Laboratory Standards Institute guidelines **CSA**: Central statistical agency **EPHI**: Ethiopian Public Health Institution **HAI**: Hospital Acquired Infections **HIV**: Human Immunodeficiency Virus **MDR**: Multi Drug Resistant **SOP**: Standard Operating Procedures **VRE**: Vancomycin Resistance *Enterococcus* **VRSA**: Vancomycin-Resistant *Staphylococcus aureus* **WHO**: World Health Organization

Commented [k59]: Deleted

Commented [k60]: Deleted

Acknowledgments

Commented [k61]: Level 1 heading, bold type, font size 18

The authors would like to thank those who were involved in this research.

Authors' contributions

Commented [k62]: Level 1 heading, bold type, font size 18

ZS, BRD, and MT carried out proposal development, data collection, data analysis and drafted the manuscript. All authors read and approved the final manuscript.

Competing interests

The authors declare that they have no competing interests

Commented [k63]: Level 1 heading, bold type, font size 18

Funding: The funders had no role in study design, data collection and analysis, decision to publish, or preparation of the manuscript. The authors received no specific funding for this work.

Commented [k64]: Financial disclosure is mentioned here as per the comment given by editor

Commented [k65]: Financial disclosure is mentioned here as per the comment given by editor

References

Commented [k66]: Level 1 heading, bold type, font size 18

1. Batistão DW da F, Gontijo-Filho PP, Conceição N, Oliveira AG de, Ribas RM (2012). Risk factors for vancomycin-resistant enterococci colonisation in critically ill patients. *Mem Inst Oswaldo Cruz*. 107(1):57–63.
2. Agegne M, Abera B, Derbie A, Yismaw G, Shiferaw MB (2018). Magnitude of Vancomycin-Resistant Enterococci (VRE) Colonization among HIV-Infected Patients Attending ART Clinic in West Amhara Government Hospitals. *Int J Microbiol*. 1–7.
3. Tacconelli E (2017). Global Priority List of Antibiotic-Resistant Bacteria to Guide Research, Discovery, and Development of New Antibiotics. WHO.
4. Ventola CL (2015). The Antibiotic Resistance Crisis: Part 1: Causes and Threats. *Pharm Ther*. 40(4):277.
5. Alotaibi FE, Bukhari EE (2017). Emergence of Vancomycin-resistant Enterococci at a Teaching Hospital, Saudi Arabia. *Chin Med J (Engl)*. 130(3):340–6.
6. Emaneini M, Hosseinkhani F, Jabalameli F, Nasiri MJ, Dadashi M, Pouriran R, et al (2016). Prevalence of vancomycin-resistant Enterococcus in Iran: a systematic review and meta-analysis. *Eur J Clin Microbiol Infect Dis*. 35(9):1387–92.
7. Alevizakos M, Gaitanidis A, Nasioudis D, Tori K, Flokas ME, Mylonakis E (2017). Colonization With Vancomycin-Resistant Enterococci and Risk for Bloodstream Infection Among Patients With Malignancy: A Systematic Review and Meta-Analysis. *Open Forum Infect Dis* [Internet]. [cited 2019 Nov 5];4(1).
8. Susan L Fraser CJD, Robert A Salata (2018). Vancomycin Resistant Enterococcal Infections: Background, Pathophysiology, Epidemiology. *Medscape*. 97(4):223–7.

9. Murray PR, Rosenthal KS, Pfaller MA (2016). Medical microbiology. 8th edition. Philadelphia, PA: *Elsevier*.
10. Nilsson O (2012). Vancomycin resistant enterococci in farm animals – occurrence and importance. *Infect Ecol Epidemiol*. 2(1):16959.
11. Sood S, Malhotra M, Das BK, Kapil A (2008). Enterococcal infections & antimicrobial resistance. *Indian J Med Res*. 128(2):111–21.
12. Crank C, O'Driscoll T (2015). Vancomycin-resistant enterococcal infections: epidemiology, clinical manifestations, and optimal management. *Infect Drug Resist.* 217.
13. Carroll KC, Hobden JA, Miller S, Morse SA, Mietzner TA, Detrick B, *et al* (2016). Jawetz, Melnick & Adelberg's medical microbiology. 27 th edition. *New York: Mcgraw-Hill Education*.
14. Faron ML, Ledeboer NA, Buchan BW (2016). Resistance Mechanisms, Epidemiology, and Approaches to Screening for Vancomycin-Resistant Enterococcus in the Health Care Setting. Kraft CS, editor. *J Clin Microbiol.* 54(10):2436–47.
15. Kateete DP, Edolu M, Kigozi E, Kisukye J, Baluku H, Mwiine FN, *et al* (2019). Species, antibiotic susceptibility profiles and van gene frequencies among enterococci isolated from patients at Mulago National Referral Hospital in Kampala, Uganda. *BMC Infect Dis*. 19(1):486.
16. Ali S, Alemayehu M, Dagnew M, Gebrecherkos T (2018). Vancomycin-Resistant Enterococci and Its Associated Risk Factors among HIV-Positive and -Negative Clients Attending Dessie Referral Hospital, Northeast Ethiopia. *Int J Microbiol.* 1–9.
17. Al-Dahmoshi HOM, Rabeea HWS, Aridhee ASA, Al-Khafaji NSK, Al-Allak MH, Lazm AM, *et al* (2019). Phenotypic Investigation of Vancomycin, Teicoplanin and Linezolid Resistance Among Enterococcus spp. Isolated from Children Diarrhea. *J Pure Appl Microbiol.* 13(1):531–6.
18. Abebe W, Endris M, Tiruneh M, Moges F. Prevalence of vancomycin resistant Enterococci and associated risk factors among clients with and without HIV in Northwest Ethiopia: a cross-sectional study. *BMC Public Health.* 2014 Dec;14(1):185.
19. Amberpet R, Sistla S, Parija SC, Rameshkumar R (2018). Risk factors for intestinal colonization with vancomycin resistant enterococci' A prospective study in a level III pediatric intensive care unit. *J Lab Physicians*. 10(1):89–94.

20. Axelrad JE, Lebwohl B, Cuaresma E, Cadwell K, Green PHR, Freedberg DE (2018). Gut colonization with vancomycin-resistant Enterococcus and risk for subsequent enteric infection. *Gut Pathog.* 10(1):28.
21. Tatsing Foka FE, Kumar A, Ateba CN (2018). Emergence of vancomycin-resistant enterococci in South Africa: Implications for public health. *South Afr J Sci* [Internet]. [cited 2019 Dec 3];114(9/10).
22. Orsi GB, Ciorba V (2013). Vancomycin resistant enterococci healthcare associated infections. *Ann Ig.* 25(10):485–92.
23. Rameshkumar MR, Arunagirinathan N (2018). Drug-Resistant Bacterial Infections in HIV Patients. *Intech Open*. 98(5).
24. Kwobah C, Ali I, Koskei P, Chumba D, Emonyi W, Mwangi A, *et al* (2015) Antibiotic Sensitivity Patterns Among Postmortem Bacterial Isolates From HIV-Infected Patients in Western Kenya: A Cross-sectional Descriptive Study. *J Dis Glob Health*. 3:73–82.
25. Chinnambedu RS, Marimuthu RR, Sunil SS, Amrose P, Ramachandran V, Pachamuthu B (2020). Changing antibiotic resistance profile of Staphylococcus aureus isolated from HIV patients (2012–2017) in Southern India. *J Infect Public Health.*13(1):75–9.
26. Remschmidt C, Schröder C, Behnke M, Gastmeier P, Geffers C, Kramer TS (2018). Continuous increase of vancomycin resistance in enterococci causing nosocomial infections in Germany − 10 years of surveillance. *Antimicrob Resist Infect Control*. 7(1):54.
27. Census 2007 Tables: Southern Peoples, Nations and Nationalities Region Archived November 13, 2012, at the Way back Machine. Tables 2.1, and 3.4.
28. Naing L, Winn T, Rusli BN (2006). Medical Statistics Practical Issues in Calculating the Sample Size for Prevalence Studies. *Archives of Orofacial Sciences* 1: 9-14.
29. Bile Esculin Azide Agar ISO Formato 30 Plates [Internet]. [cited 2020 Jan 24].
30. CLSI (2018) Clinical and Laboratory Standards Institute, Performance Standards for Antimicrobial Susceptibility Testing, CLSI, Wayne, PA, USA, 28th edition.
31. Ferede ZT, Tullu KD, Derese SG, Yeshanew AG (2018). Prevalence and antimicrobial susceptibility pattern of Enterococcus species isolated from different clinical samples at Black Lion Specialized Teaching Hospital, Addis Ababa, Ethiopia. *BMC Res Note*s 11(793). DOI: 10.1186/s13104-018-3898-0
32. Markwart R, Willrich N, Haller S, Noll I, Koppe U, Werner G, *et al* (2019). The rise in vancomycin-resistant Enterococcus faecium in Germany: data from the German

Antimicrobial Resistance Surveillance (ARS). *Antimicrob Resist Infect Control*. 8(1):1–11.

33. Deshpande VR, Karmarkar MG, Mehta PR (2013). Prevalence of multidrug-resistant enterococci in a tertiary care hospital in Mumbai, India. *J Infect Dev Ctries*. 7(02):155–8.
34. Flokas ME, Karageorgos SA, Detsis M, Alevizakos M, Mylonakis E (2017). Vancomycin-resistant enterococci colonisation, risk factors and risk for infection among hospitalised paediatric patients: a systematic review and meta-analysis. *Int J Antimicrob Agents*. 49(5):565–72.
35. Cattoir V, Leclercq R (2013). Twenty-five years of shared life with vancomycin-resistant enterococci: is it time to divorce? *J Antimicrob Chemother*. 68(4):731–42.
36. Achenbach C, Flores E, Ferrell P, Pitrak D, Weber SG (2006). Prevalence of and Risk Factors for Colonization With Vancomycin-Resistant Enterococcus Among Human Immunodeficiency Virus–Positive Outpatients. *Infect Control Hosp Epidemiol*. 27(1):102–4.
37. Ekuma AE, Oduyebo OO, Efunshile AM, Konig B (2016). Surveillance for vancomycin resistant enterococci in a tertiary institution in south western Nigeria. *Afr J Infect Dis.* 10(2):121–6.
38. Swissnoso, Buetti N, Wassilew N, Rion V, Senn L, Gardiol C, *et al* (2019) Emergence of vancomycin-resistant enterococci in Switzerland: a nation-wide survey. *Antimicrob Resist Infect Control.* 8(1):16.
39. Yilema A, Moges F, Tadele S, Endris M, Kassu A, Abebe W, *et al* (2017) Isolation of enterococci, their antimicrobial susceptibility patterns and associated factors among patients attending at the University of Gondar Teaching Hospital. *BMC Infect Dis.* 17(1):276.
40. Abamecha A, Wondafrash B, Abdissa A (2015). Antimicrobial resistance profile of Enterococcus species isolated from intestinal tracts of hospitalized patients in Jimma, Ethiopia. *BMC Res Notes*. 8(1):213.
41. Toru M, Beyene G, Kassa T, Gizachew Z, Howe R, Yeshitila B (2018). Prevalence and phenotypic characterization of Enterococcus species isolated from clinical samples of pediatric patients in Jimma University Specialized Hospital, south west Ethiopia. *BMC Res Notes*. 11(1):281.
42. Anvarinejad M, Pouladfar G, Japoni A, Bolandparvaz S, Satiary Z, Mardaneh J (2016). Diabetic Foot Infections: Antibiotic Susceptibility Patterns and Determination of

Antibiotic Cross-Resistance in Clinical Isolates of Enterococcus Species During 2012 - 2014 in Shiraz, Iran. *Arch Pediatr Infect Dis*. 5(2).

43. Kee SY, Park CW, Lee JE, Kwon YJ, Pyo HJ, *et al.*(2012) Healthcare-associated risk factors of vancomycin-resistant Enterococci colonization among outpatients undergoing hemodialysis. *Jpn J Infect Dis*. 65(1):57–60.
44. Moemen D, Tawfeek D, Badawy W (2015). Healthcare-associated vancomycin resistant Enterococcus faecium infections in the Mansoura University Hospitals intensive care units, Egypt. *Braz J Microbiol.* 46(3):777–83.

Figure legends

Figure 1 Prevalence of Vancomycin Resistant *Enterococci* among HIV Infected Patients on ART at Arba Minch General Hospital, Southern Ethiopia, 2020 (n=123)

Figure 2 Antimicrobial Resistance Patterns of Isolated *Enterococcus* spp among HIV Infected Patients on ART at Arba Minch General Hospital, Southern Ethiopia, 2020 (n=123)

Commented [k67]: Deleted

Point by point response

1. Response to editors

Comment given: Please ensure that your manuscript meets PLOS ONE's style requirements, including those for file naming. The PLOS ONE style templates can be found at https://journals.plos.org/plosone/s/file?id=wjVg/PLOSOne_formatting_sample_main_body.pdf andhttps://journals.plos.org/plosone/s/file?id=ba62/PLOSOne_formatting_sample_title_authors_affiliations.pdf

Response: Comment accepted and corrections were made following the guideline and sample template. (Check for tracking documents where changes made accordingly)

Comment given: Please include additional information regarding the survey or questionnaire used in the study and ensure that you have provided sufficient details that others could replicate the analyses. For instance, if you developed a questionnaire as part of this study and it is not under a copyright more restrictive than CC-BY, please include a copy, in both the original language and English, as Supporting Information.

Response: Comment accepted and questionnaire (both in local language and English) included as additional information.

Comment given: Thank you for stating the following financial disclosure:

> "The funders had no role in study design, data collection and analysis, decision to publish, or preparation of the manuscript."

At this time, please address the following queries:

a. Please clarify the sources of funding (financial or material support) for your study. List the grants or organizations that supported your study, including funding received from your institution.
b. State what role the funders took in the study. If the funders had no role in your study, please state: "The funders had no role in study design, data collection and analysis, decision to publish, or preparation of the manuscript."
c. If any authors received a salary from any of your funders, please state which authors and which funders.
d. If you did not receive any funding for this study, please state: "The authors received no specific funding for this work."

Response: Comment accepted financial disclosure mentioned in the manuscript "The funders had no role in study design, data collection and analysis, decision to publish, or preparation of

the manuscript. The authors received no specific funding for this work" (see page 17 paragraph 1)

Comment given: We note that you have indicated that data from this study are available upon request. PLOS only allows data to be available upon request if there are legal or ethical restrictions on sharing data publicly. In your revised cover letter, please address the following prompts: a) If there are ethical or legal restrictions on sharing a de-identified data set, please explain them in detail (e.g., data contain potentially identifying or sensitive patient information) and who has imposed them (e.g., an ethics committee). Please also provide contact information for a data access committee, ethics committee, or other institutional body to which data requests may be sent.

Response: We have stated that data from this study are available upon request. This is due to Ethical issues (data contain sensitive patient information that cannot be disclosed and can be used for research only). The data can be found upon reasonable request from corresponding author (belayjanimen@gmail.com).

Comment given: PLOS requires an ORCID iD for the corresponding author in Editorial Manager on papers submitted after December 6th, 2016. Please ensure that you have an ORCID iD and that it is validated in Editorial Manager. To do this, go to 'Update my Information' (in the upper left-hand corner of the main menu), and click on the Fetch/Validate link next to the ORCID field. This will take you to the ORCID site and allow you to create a new iD or authenticate a pre-existing iD in Editorial Manager.

Response: Comment accepted.

Comment given: Your ethics statement should only appear in the Methods section of your manuscript. If your ethics statement is written in any section besides the Methods, please move it to the Methods section and delete it from any other section. Please ensure that your ethics statement is included in your manuscript, as the ethics statement entered into the online submission form will not be published alongside your manuscript.

Response: Comment accepted and ethics section moved to Methods section (see page 8 Ethics approval section)

Comment given: We note you have included a table to which you do not refer in the text of your manuscript. Please ensure that you refer to Table 5 in your text; if accepted, production will need this reference to link the reader to the Table.

Response: Table 5 is already mentioned on page 11 in line with Table 4 [Table 4 and 5] (on section "Factors associated with vancomycin resistant *Enterococcus* gut colonization")

Comment given: Please include your tables as part of your main manuscript and remove the individual files. Please note that supplementary tables (should remain/ be uploaded) as separate "supporting information" files.

Response: Comment accepted and all Tables included in the manuscript (see page 8-12 for Table 1-5)

Comment given: Please include captions for your Supporting Information files at the end of your manuscript, and update any in-text citations to match accordingly.

Response: Comment accepted.

Comment given: The reviewers have commented on the manuscript and raised number of pertinent points that need to be addressed. Discussion section should interpret the results and discuss in the light of current knowledge rather than repeating the results. This applies to Conclusion section abstract too. Please revise the manuscript addressing all reviewer comments.

Response: Comment accepted and revisions were made according to comment give by reviewers and see the responses given below:-

2. Response to Reviewers

Comment given: The language should be improved.

Response: We tried to amend and also re-write some parts of the manuscript. Check the manuscript again [please check for Abstract, Introduction, Materials and Methods, Result, Discussion, Conclusion and Limitations of the study sections.]

Comment given: The manuscript presents extensive introduction which can be reduced to two or three paragraphs.

Response: Comment accepted and Introduction section reduced to three paragraphs on the manuscript (see page 2)

Comment given: Statistical tests should be mentioned in the abstract also.

Response: Comment accepted and statistical tests added to Abstract section ("Bivariable and multivariable logistic regression model was used to analyze the association between dependent and independent variables. P-values in the multivariable analysis, adjusted odds ratio (AOR) and 95% confidence interval (CI) were used to determine the strength of association. P-value $\leq$0.05 was considered as significant.")

Comment given: In methodology, some sentences can be eliminated like the section on antimicrobial susceptibility testing.

Response: Comment accepted and antimicrobial susceptibility testing deleted (page 4)

Comment given: The name of the institute should be masked.

Response: It is impossible to mask the name of the institute where research was conducted. If we mask the name of the institute where the research is conducted, the research will not be trustworthy to the scientific world.

Comment given: Discussion needs to be improved. Rather than stating the facts, the results should be compared and justification (or hypotheses) for any differences should be explored.

Response: Comment accepted and discussion section revised according to the comments.

Comment given: The disk diffusion test alone was used. Explain why MIC was not estimated and species level identification was not performed.

Response: Due to limitation of resources and budget, species level identification and MIC were not performed (see page 13 "Limitations of the study" section.)
