# Supplementary material for: Vancomycin resistant Enterococci and its associated factors among HIV infected patients on anti-retroviral therapy in Ethiopia
Source: PLoS One. 2021 Jun 24;16(6):e0251727. doi: 10.1371/journal.pone.0251727 (PMC8224944; doi:10.1371/journal.pone.0251727)
Supplement: Attachment — Submitted filename: Point by point response.docx [file pone.0251727.s003.docx]

**Point by point response**

1. **Response to editors**

**Comment given**: Please ensure that your manuscript meets PLOS ONE's style requirements, including those for file naming. The PLOS ONE style templates can be found at <https://journals.plos.org/plosone/s/file?id=wjVg/PLOSOne_formatting_sample_main_body.pdf>  and<https://journals.plos.org/plosone/s/file?id=ba62/PLOSOne_formatting_sample_title_authors_affiliations.pdf>

**Response**: Comment accepted and corrections were made following the guideline and sample template. (Check for tracking documents where changes made accordingly)

**Comment given**: Please include additional information regarding the survey or questionnaire used in the study and ensure that you have provided sufficient details that others could replicate the analyses. For instance, if you developed a questionnaire as part of this study and it is not under a copyright more restrictive than CC-BY, please include a copy, in both the original language and English, as Supporting Information.

**Response**: Comment accepted and questionnaire (both in local language and English) included as additional information.

**Comment given**: Thank you for stating the following financial disclosure:

"The funders had no role in study design, data collection and analysis, decision to publish, or preparation of the manuscript."

At this time, please address the following queries:

a. Please clarify the sources of funding (financial or material support) for your study. List the grants or organizations that supported your study, including funding received from your institution.
b. State what role the funders took in the study. If the funders had no role in your study, please state: “The funders had no role in study design, data collection and analysis, decision to publish, or preparation of the manuscript.”
c. If any authors received a salary from any of your funders, please state which authors and which funders.
d. If you did not receive any funding for this study, please state: “The authors received no specific funding for this work.”

**Response**: Comment accepted financial disclosure mentioned in the manuscript “The funders had no role in study design, data collection and analysis, decision to publish, or preparation of the manuscript. The authors received no specific funding for this work” (see page 17 paragraph 1)

**Comment given**: We note that you have indicated that data from this study are available upon request. PLOS only allows data to be available upon request if there are legal or ethical restrictions on sharing data publicly. In your revised cover letter, please address the following prompts: a) If there are ethical or legal restrictions on sharing a de-identified data set, please explain them in detail (e.g., data contain potentially identifying or sensitive patient information) and who has imposed them (e.g., an ethics committee). Please also provide contact information for a data access committee, ethics committee, or other institutional body to which data requests may be sent.

**Response**: We have stated that data from this study are available upon request. This is due to Ethical issues (data contain sensitive patient information that cannot be disclosed and can be used for research only). The data can be found upon reasonable request from corresponding author ([belayjanimen@gmail.com](mailto:belayjanimen@gmail.com)).

**Comment given**: PLOS requires an ORCID iD for the corresponding author in Editorial Manager on papers submitted after December 6th, 2016. Please ensure that you have an ORCID iD and that it is validated in Editorial Manager. To do this, go to ‘Update my Information’ (in the upper left-hand corner of the main menu), and click on the Fetch/Validate link next to the ORCID field. This will take you to the ORCID site and allow you to create a new iD or authenticate a pre-existing iD in Editorial Manager.

**Response**: Comment accepted.

**Comment given**: Your ethics statement should only appear in the Methods section of your manuscript. If your ethics statement is written in any section besides the Methods, please move it to the Methods section and delete it from any other section. Please ensure that your ethics statement is included in your manuscript, as the ethics statement entered into the online submission form will not be published alongside your manuscript.

**Response**: Comment accepted and ethics section moved to Methods section (see page 8 Ethics approval section)

**Comment given**: We note you have included a table to which you do not refer in the text of your manuscript. Please ensure that you refer to Table 5 in your text; if accepted, production will need this reference to link the reader to the Table.

## Response: Table 5 is already mentioned on page 11 in line with Table 4 [Table 4 and 5] (on section “Factors associated with vancomycin resistant *Enterococcus* gut colonization”)

**Comment given**: Please include your tables as part of your main manuscript and remove the individual files. Please note that supplementary tables (should remain/ be uploaded) as separate "supporting information" files.

**Response**: Comment accepted and all Tables included in the manuscript (see page 8-12 for Table 1-5)

**Comment given**: Please include captions for your Supporting Information files at the end of your manuscript, and update any in-text citations to match accordingly.

**Response**: Comment accepted.

**Comment given**: The reviewers have commented on the manuscript and raised number of pertinent points that need to be addressed. Discussion section should interpret the results and discuss in the light of current knowledge rather than repeating the results. This applies to Conclusion section abstract too. Please revise the manuscript addressing all reviewer comments.

**Response**: Comment accepted and revisions were made according to comment give by reviewers and see the responses given below:-

2. **Response to Reviewers**

**Comment given**: The language should be improved.

**Response**: We tried to amend and also re-write some parts of the manuscript. Check the manuscript again [please check for Abstract, Introduction, Materials and Methods, Result, Discussion, Conclusion and Limitations of the study sections.]

**Comment given**: The manuscript presents extensive introduction which can be reduced to two or three paragraphs.

**Response**: Comment accepted and Introduction section reduced to three paragraphs on the manuscript (see page 2)

**Comment given**: Statistical tests should be mentioned in the abstract also.

**Response**: Comment accepted and statistical tests added to Abstract section (“Bivariable and multivariable logistic regression model was used to analyze the association between dependent and independent variables. P-values in the multivariable analysis, adjusted odds ratio (AOR) and 95% confidence interval (CI) were used to determine the strength of association. P-value ≤0.05 was considered as significant.”)

**Comment given**: In methodology, some sentences can be eliminated like the section on antimicrobial susceptibility testing.

**Response**: Comment accepted and antimicrobial susceptibility testing deleted (page 4)

**Comment given**: The name of the institute should be masked.

**Response**: It is impossible to mask the name of the institute where research was conducted. If we mask the name of the institute where the research is conducted, the research will not be trustworthy to the scientific world.

**Comment given**: Discussion needs to be improved. Rather than stating the facts, the results should be compared and justification (or hypotheses) for any differences should be explored.

**Response**: Comment accepted and discussion section revised according to the comments.

**Comment given**: The disk diffusion test alone was used. Explain why MIC was not estimated and species level identification was not performed.

**Response**: Due to limitation of resources and budget, species level identification and MIC were not performed (see page 13 “Limitations of the study” section.)
