# Supplementary material for: Vancomycin resistant Enterococci and its associated factors among HIV infected patients on anti-retroviral therapy in Ethiopia
Source: PLoS One. 2021 Jun 24;16(6):e0251727. doi: 10.1371/journal.pone.0251727 (PMC8224944; doi:10.1371/journal.pone.0251727)
Supplement: Attachment — Submitted filename: Point by point response.docx [file pone.0251727.s005.docx]

**Point by point response**

**Comment given**: Journal Requirements: Please review your reference list to ensure that it is complete and correct. If you have cited papers that have been retracted, please include the rationale for doing so in the manuscript text, or remove these references and replace them with relevant current references. Any changes to the reference list should be mentioned in the rebuttal letter that accompanies your revised manuscript. If you need to cite a retracted article, indicate the article’s retracted status in the References list and also include a citation and full reference for the retraction notice.

**Response**: Comment accepted and corrections were made on all references. (Check for tracking documents where changes were made on reference section, page 15-19)

**Comment given**: Table 3: G,P,TTC,MAC - all isolates are resistant to these groups. The table is conveying wrong information. .

**Response**: Comment accepted and correction made on the Table 3. All isolates are resistant to G, P, TTC, MAC 14 (100%); (G, P, TTC, MAC) + F 2 (14.28%) and (G, P, TTC, MAC) + F+ PH 11 (78.58%) (please check Table 3 page 9)

**Comment given**: It is unclear whether these are the studies conducted on HIV infected patients or not. If not, mention it. (Discussion section on page 11-12 paragraph 1)

**Response**: Comment accepted and correction made on the manuscript by declaring HIV status of studies done in different area (see page 11-12 paragraph 1 of Discussion section)

**Comment given**: No italics or upper case for “*Enterococci”* (page 13 paragraph 2).

**Response**: Comment accepted and re-written as Enterococci (please check on revised manuscript page 13 paragraph 2)

**Comment given**: correct as “were not done” on Limitation of study section which was written previously as:- “The isolated *Enterococci* were not identified to species level; molecular characterization and MIC **were done** due to resource limitation and budget constraints”

**Response**: Comment accepted and “were done” changed to “were not done” in limitation of the study section and re-written as “The isolated *Enterococci* were not identified to species level; molecular characterization and MIC **were not done** due to resource limitation and budget constraints”

**Comment given**: Conclusion section paragraph 2 “Therefore periodic surveillance on antimicrobial resistance pattern of *Enterococcus* species is important for proper treatment, health professionals should strictly follow infection prevention protocols and further studies should be conducted on species identification and molecular characterization of *Enterococci*” marked as “Not a part of conclusion” by Reviewer

.

**Response**: Comment accepted and re-written as “Therefore, periodic surveillance on antimicrobial resistance pattern, adhering to rational use of antibiotics and implementing infection prevention protocols may reduce colonization by VRE” (see page 14 Conclusion section paragraph 2)

**Comment given**: Figure 1 not necessary as it can be expressed in text.

## Response: Comment accepted and Figure 1 is expressed in words in Result section Incidence of vancomycin resistant *Enterococcus* sub section on page 8

**Comment given**: Figure 2 change ug symbol

**Response**: Comment accepted and ug symbol is changed to µg.

**Comment given**: Have the authors made all data underlying the findings in their manuscript fully available? The [PLOS Data policy](http://www.plosone.org/static/policies.action#sharing) requires authors to make all data underlying the findings described in their manuscript fully available without restriction, with rare exception (please refer to the Data Availability Statement in the manuscript PDF file). The data should be provided as part of the manuscript or its supporting information, or deposited to a public repository. For example, in addition to summary statistics, the data points behind means, medians and variance measures should be available. If there are restrictions on publicly sharing data—e.g. participant privacy or use of data from a third party—those must be specified.

Reviewer #1: No

**Response**: Comment accepted and **Data availability statement** added to page 14 that say “Data cannot be shared publicly because of ethical issues. However the data underlying the results presented in the study are available from corresponding author (belayjanimen@gmail.com) and Institutional Review Board (IRB) committee /Zerihun Zerdo (zedozerihun@gmail.com) on reasonable request.” (please check page 14 **Data availability statement** section**)**

**Comment given**: Remove tables from main text if presented at the end of the manuscript

**Response**: Tables are submitted in the main text only neither submitted individually nor the end of the manuscript
